# In Silico Modeling of the Influence of Environment on Amyloid Folding Using FOD-M Model

**DOI:** 10.3390/ijms221910587

**Published:** 2021-09-30

**Authors:** Irena Roterman, Katarzyna Stapor, Piotr Fabian, Leszek Konieczny

**Affiliations:** 1 Department of Bioinformatics and Telemedicine, Medical College, Jagiellonian University, Medyczna 7, 30-688 Kraków, Poland; 2 Institute of Computer Science, Silesian University of Technology, Akademicka 16, 44-100 Gliwice, Poland; katarzyna.stapor@polsl.pl (K.S.); piotr.fabian@polsl.pl (P.F.); 3 Chair of Medical Biochemistry, Medical College, Jagiellonian University, Kopernika 7, 31-034 Kraków, Poland; mbkoniec@cyf-kr.edu.pl

**Keywords:** hydrophobicity, amyloid, water environment, micelle, membrane environment

## Abstract

The role of the environment in amyloid formation based on the fuzzy oil drop model (FOD) is discussed here. This model assumes that the hydrophobicity distribution within a globular protein is consistent with a 3D Gaussian (3DG) distribution. Such a distribution is interpreted as the idealized effect of the presence of a polar solvent—water. A chain with a sequence of amino acids (which are bipolar molecules) determined by evolution recreates a micelle-like structure with varying accuracy. The membrane, which is a specific environment with opposite characteristics to the polar aquatic environment, directs the hydrophobic residues towards the surface. The modification of the FOD model to the FOD-M form takes into account the specificity of the cell membrane. It consists in “inverting” the 3DG distribution (complementing the Gaussian distribution), which expresses the exposure of hydrophobic residues on the surface. It turns out that the influence of the environment for any protein (soluble or membrane-anchored) is the result of a consensus factor expressing the participation of the polar environment and the “inverted” environment. The ratio between the proportion of the aqueous and the “reversed” environment turns out to be a characteristic property of a given protein, including amyloid protein in particular. The structure of amyloid proteins has been characterized in the context of prion, intrinsically disordered, and other non-complexing proteins to cover a wider spectrum of molecules with the given characteristics based on the FOD-M model.

## 1. Introduction

The issue of neurodegenerative diseases is obviously related to amyloids [1,2]. In this discussion, one should distinguish the phenomenon of amyloidosis occurring in the human body [3,4,5,6,7,8] compared to in vitro experiments [9,10,11,12,13,14,15,16]. The main point of many works is the recognition of the mechanism of amyloid transformation [17,18,19,20]. One of the proposed approaches links amyloid transformation with intrinsically disordered proteins [21,22,23,24,25,26]. Experiments identify external factors favoring the amyloid transformation [27]. The most interesting factor among them is the purely physical one—shaking [28]. The specificity of the air–water interphase implies certain phenomena [29,30,31,32] to which the folding protein is subjected. This method of obtaining amyloids eliminates chemical factors, focusing solely on the physical phenomenon of dissolving air in water significantly increasing the presence of air–water interphase. The presence of air in water affects the structuring of water, which under the influence of this factor changes its standard order—so far unknown. Research on the relationship between water and hydrophobic surfaces is of particular importance here [33]. Shaking as a technique to generate amyloid structures in in vitro techniques does not appear to be a factor observed in vivo. This observation suggests that amyloid transformation can occur according to at least two scenarios. It seems that in the case of transformation taking place under physiological conditions, the factors favoring this process are chemical factors such as pH change and the presence of other compounds, which are summarized in [27]. It is assumed that these factors affect the structurization of water, and thus the form of the external force field, which affects the very process of folding polypeptide chains and the possible change in conformation [34]. The polar environment, minimizing the adverse entropy effect associated with the interaction with hydrophobic systems, directs hydrophobic residues towards the central part of the protein molecule with simultaneous exposure of polar residues on the surface. This leads to an arrangement similar to that seen for the spherical micelle structure. The hydrophobicity distribution in the protein can therefore be described by the 3D Gaussian distribution (3DG), where the maximum is located in the center, while the hydrophobicity density decreases closer to the surface. 

Numerous groups of proteins have been identified that represent the hydrophobicity distribution consistent with the proposed function. These are down-hill, fast-folding, ultra-fast-folding, and antifreeze type II and III proteins [35]. Proteins whose chains with the evolutionarily determined amino acid sequence are not able to generate a structure with a distribution consistent with the distribution of 3DG have also been identified. This maladjustment may take a local form: the local excess of hydrophobicity on the surface determines the site of potential interaction with another protein [36], while the local deficiency is usually associated with the presence of a cavity, which turns out to be the site of ligand complexation, or in the case of enzymes, substrates [36].

The chemically opposite environment for proteins is the membrane environment which does not follow the 3DG distribution. In the analysis of membrane proteins, the FOD model taking into account the modification of FOD-M is used, where the membrane contribution is expressed by the complement function expressed as 1-3DG. It expresses the inverse distribution to the system observed in soluble proteins. This is the case with membrane proteins which additionally play the role of ion channels, where apart from the exposure of the hydrophobic surface (directed towards the environment of the membrane), polar residues forming the surface of the channel are located in the center [37].

The environment that guarantees the activity of proteins is not pure water (0.9% NaCl) or a pure hydrophobic environment—a membrane. Therefore, usually the environment for a protein is the result of a consensus between the participation of the polar water environment, but also the presence of numerous molecules with a polarity other than that of water. The degree of mismatch in the hydrophobicity distribution in the protein may suggest the participation of factors involved in the process of shaping the structure of a given protein. Such a consensus, expressed in the quantitative contribution of the polar environment and the opposite to polar environment, was applied to the exemplary membrane proteins serving as the ion channel and to proteins of different status [37]. Generally speaking, such a quantitative assessment of the differences between the idealized (3DG) distribution and that observed in the protein (the result of inter-amino-acid interactions) allows speculation about the participation of a factor other than water in the process of shaping the structure. In the discussed model, the hydrophobicity distribution resulting from the influence of the polar environment is expressed in the protein molecule by means of the 3D Gaussian function with a centrally located hydrophobic core. The membrane environment for stable interaction with the protein expects a hydrophobic surface of the protein. If, in addition, the central part plays the role of a channel (ion or efflux transport), unlike a soluble protein with a hydrophobic core, the central part represents a low level of hydrophobicity or even a polar environment. Therefore, the function (1-3DG) is used to describe such proteins. It turns out, however, that the widespread presence of water in living organisms contributes to the formation of proteins fully anchored in the membrane. Therefore, the presence of both these environments should be taken into account in the description of protein structures, including membrane structures. A thorough analysis of randomly selected proteins suggests that the consensus between the participation of the aqueous and non-polar environment concerns the vast majority of proteins. However, the relationship is very diverse: from the dominant 3DG-type system with a small part of (1-3DG) to the inverse situation. In the discussed model, this relationship is expressed by the parameter K, which strengthens or weakens the amount of the hydrophobic factor. Hence, the mentioned down-hill and fast-folding proteins represent a status which is the effect of the influence of the aquatic environment only at K = 0. The structure of these proteins represents the hydrophobicity distribution and fully reflects the 3DG system. Large K values are identified in the case of membrane proteins or proteins that require a specific “frame” (also referred to as a permanent chaperone) to fix their structure. Such an example is discussed in this paper. The present work discusses changes in the status of protein structure undergoing amyloid transformation, and these changes are expressed and “traced” by the value of the parameter K.

On the basis of the analysis of such proteins (unfortunately, it is not a large group), one can speculate as to the structural changes of other amyloids with the only known structure of the amyloid form [38,39,40,41,42,43,44,45,46,47,48]. The FOD-M modification presented in detail in [49] used to describe the spectrum of proteins differing in the degree of hydrophobicity order in relation to the assumed 3D Gaussian distribution justifies the use of this model for protein description. Since the introduced model, aimed at the interpretation of the specific structure of amyloids, requires interpretation in terms of a wider spectrum of protein structures, the analysis was extended to include the group of other amyloids [50,51,52,53,54,55,56,57,58]: prion proteins were included [59,60,61,62,63,64,65,66,67], a group of proteins identified as intrinsically disordered due to their discussed predisposition to amyloid transformation [68,69,70,71,72,73,74], and short peptides both in their monomeric form and in the form of complexes [75,76,77,78,79,80,81,82,83,84,85,86,87]. In order not to limit the proteins directly or indirectly related to the phenomenon of amyloidosis, the spectrum of the analyzed proteins was extended to include non-complexing proteins [88,89,90,91,92,93,94,95], a representative of enzymes [96,97], and a transmembrane protein [98]. The latter are discussed in detail in [37,49]. These lists do not exhaust the full range of the protein groups mentioned. The given examples reveal the differentiation of status in terms of the fuzzy oil drop model and present this model as a universal tool enabling its application to the analysis of any protein.

The present work is not a review work, therefore other mechanisms of the amyloid transformation have only been shortly presented in the introduction section. The work is limited to a proposal for the interpretation of the amyloid transformation phenomenon as an effect of the environmental influence expressed by a mathematical model quantifying the contribution of factors expressing the characteristics of the environment to achieve the appropriate structure of the amyloid fibril. In order to broaden the spectrum of features of the analyzed proteins postulated as favorable for the amyloid transformation process, the analysis of prion proteins, proteins classified as intrinsically disordered short peptides with a tendency to fibrillarization, was added, and the list of available amyloid structures was extended, as was mentioned above. These additional examples, including also the proteins that do not show a tendency to complex, are intended to enable a wide range comparative analysis. An example of a transmembrane protein was also discussed in order to demonstrate its predominant hydrophobicity distribution characteristic in relation to soluble globular proteins. The set of proteins should help to identify the distinction of proteins fully accordant with the 3DG-based model: proteins with local-aim-oriented discordance between idealized and observed hydrophobicity distribution and those representing intentional-biological-activity-related global discordance. The last ones are membrane proteins accordant with the (1-3DG) distribution rather than with the 3DG one.

## 2. Results

A given protein is described by the following parameters. The parameter RD (Relative Distance) is defined for the relation of distributions T (theoretical—being the 3D Gaussian (3DG) function), O (observed), and R (uniform). The idealized theoretical 3DG distribution expresses the influence of the water environment directing structuring towards the generation of a centric hydrophobic core. The introduced distribution described by the “complement” function in the form (1-3DG) represents the hydrophobic membrane environment, where exposure of the hydrophobic residues is expected. Since the relationship between the factors 3DG and (1-3DG) is variable and characteristic for a given protein, parameter K reveals the degree of modification of the 3DG distribution with (1-3DG) distribution. As a result, the modified distribution M determines the form of the distribution—with the appropriate selection of the parameter K—which is the “target” for the distribution O. The parameter RD for the M-O-T relation determines the degree of compatibility of the distribution O and the modified distribution M in relation to the idealized distribution T. In other words, the comparison of RD for the relation of T-O-R with the value of RD for the relation M-O-T informs us about the degree of the necessary modification of the distribution T and measures the distance of the distribution O to the distribution M with a reference distribution T. The M distribution represents the modified target idealized distribution expressing the specific conditions for folding of the chain built by bi-polar molecules. The form of the M distribution is the effect of polar and non-polar compounds participating in the external force field construction. The degree of modification is expressed by the K parameter. All the parameters mentioned are discussed in detail in the section Materials and Methods.

### 2.1. Comparative Analysis of Amyloid Proteins and Their Native Forms

In the present work, determining the value of the K parameter for proteins in the form of WT and in their amyloid versions allows for the assessment of the influence of external factors (chemical, physical) on shaping the hydrophobicity distribution. It is assumed that the K value determines the specificity of the environment in which a given protein obtains a biologically active form or takes a form deviating from WT. The analysis of four proteins with different status expressed by the value of the RD parameter indicated the optimal K values for mapping the O distribution with an appropriately modified T distribution (referred to as M). Characteristics of these proteins for single chains are regarded as structural units (Table 1). Analogous characteristics of chains present in amyloid fibrils regarded as components of proto- and super-fibrils are also presented.

The value of Kullback–Leibler divergence entropy (described in detail in Section 4 Materials and Methods) depends on the number of residues in a polypeptide chain. This is why it can be applied for comparison of polypeptide chains of equal length, and why it can be used for the definition of an optimal K value individually for the polypeptide chains under consideration.

**Figure 1:**
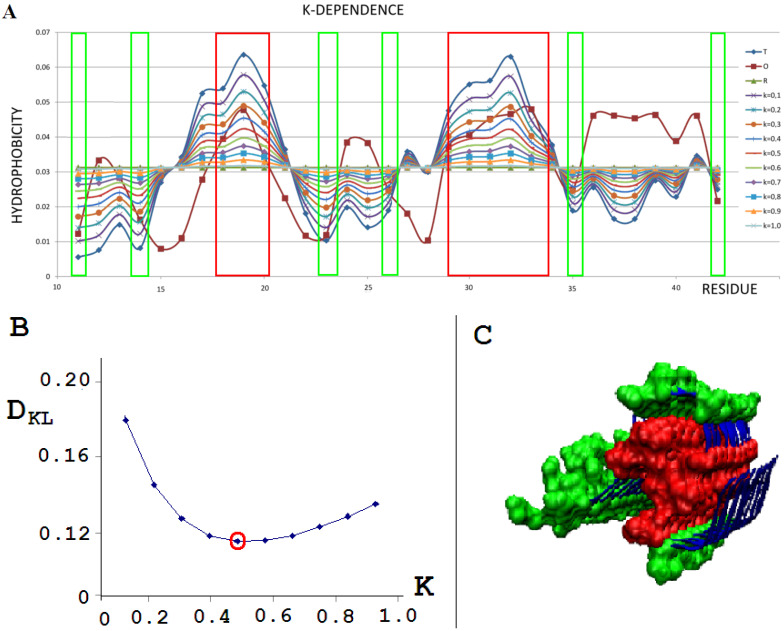
External force field analysis for a single-chain Aβ-amyloid(11–42) (PDB ID 5KK3) [38]. (**A**)—Sample of 10 distributions for different values of K: T - blue; O - brown; R - green, all other colors as shown in legend represent the modification of T distribution by different K values. (**B**)—D_KL_ dependence on K to find the lowest D_KL_ between modified T and O distribution. (**C**)—3D presentation of protofilament of Aβ-amyloid(11–42). Red residues (space filling), high hydrophobicity accordant with the T profile; green residues (space filling), low hydrophobicity accordant with the T distribution. Fragments distinguished according to frames given in A.

To illustrate the procedure determining the optimal K parameter for a given protein, the protein Aβ-amyloid was selected (Figure 1A). The profiles of M distributions with a different degree of modification of T distribution (i.e., different values of the parameter K) are shown in Figure 1.

The set of profiles (Figure 1A) visualizes the T distribution—the value K = 0 (i.e., the state where it is assumed that the structure generates the form of micelles as a response to environmental influences). This distribution deviates from the O distribution, which is often present in numerous proteins. The successive increasing values of parameter K cause the modifications of the T profile, providing the corresponding M profiles for those various values of K. Figure 1B shows the change in divergence entropy for the successive states with a different part of the component (1-3DG). It turns out that the optimal value of K approaching the distributions O and M (originally T) is the value K = 0.5. This means that the best fit of the distribution O is obtained for this value of K (i.e., this value expresses the required modification of the target distribution). If the environment expressed an influence such as the distribution M for the value of parameter K = 0.5, the structure of the chain in question would recreate this target structural form. Therefore, the influence of the environment on the structure formation is expressed by the participation of hydrophobic factors at the level of 0.5 (see Equation (6)). The brown line (Figure 1A) visualizes the O distribution. As can be seen, some chain fragments represent discordance with respect to the T profile. Chain fragments of accordant (O versus T) status—as shown in the 3D presentation (Figure 1C)—reveal the presence of a hydrophobic core (red) and polar surface (green); however, this is in limited degree, which is comparable to other proteins (except down-hill, fast-folding, and some antifreeze type III proteins, as shown in Supplementary Materials). Despite the presence of chain fragments of the discordant status (O versus T), the presence of a hydrophobic core can be seen in the structure of proto-fibril of Aβ(11–42) amyloid (PDB ID 5KK3).

### 2.2. Proteins with Increasing K Values Accompanying Amyloid Transformation

The values summarized in Table 2 clearly distinguish between the two scenarios. According to one of them, a significant increase in the K value when switching from the native form to the amyloid fibril form for both transthyretin and the V domain of the IgG light chain is the result of the introduction of an external factor in the form of a change in the structuring of the water environment (shaking or the presence of chemical factors). According to the interpretation based on the fuzzy oil drop model, the influence of the presence of chemical factors is not based on the direct effect of these factors on the protein. It is the change in the structure of the water that acts as an external force field that results in the achievement of a structural form that probably corresponds to the changed strength of the impact of the water environment on the folding protein. Increasing the value of the K parameter expresses an increase in the factor disturbing the standard effect of the water environment on the protein structure.

**Figure 2:**
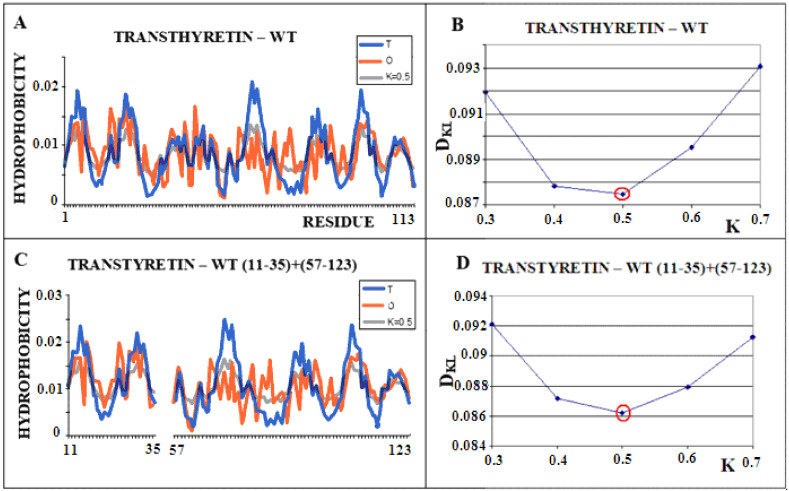
Structural characteristics of transthyretin. (**A**)—T-navy blue, O-red, and M-gray profiles for the complete chain. The M distribution is obtained for the parameter K = 0.5 according to B. (**B**)—Change in the *D_KL_* value for the O–M relation with different values of the K parameter. The lowest value was indicated as representing the shortest distance between the distribution of O and M. (**C**)—T-navy blue, O-red, and M-gray profiles for WT form of transthyretin however limited to fragments present in the amyloid form of this protein. The M distribution is obtained for the parameter K = 0.5. according to D. (**D**)—Change in the *D_KL_* value for the O–M relation with different values of the K parameter. The lowest value was indicated as representing the shortest distance between the distribution of O and M.

**Figure 3:**
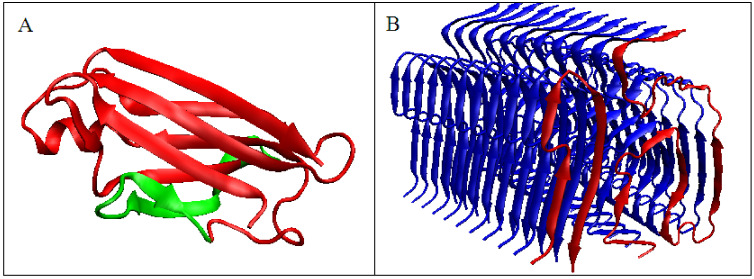
3D presentation of transthyretin chain. (**A**)—Native form, the red fragment present in amyloid form, green fragment, absent in amyloid form (**B**)—red chain, amyloid form – analog to red fragment in A.

Figure 2 shows the steps of determining the K parameter value for the native form of transthyretin (Figure 2A,B). The same operation was performed on a selected portion of the transthyretin chain present in amyloid form (fragments 11–35, 57–123). Both forms of this protein (single chain) show the most optimal adjustment of the O distribution with a parameter of K = 0.5. This means that the changed environment with the “strength” for K = 0.5 resulted in the shaping of the tertiary structure of this protein. Figure 3 visualizes the discussed fragments in 3D presentation.

**Figure 4:**
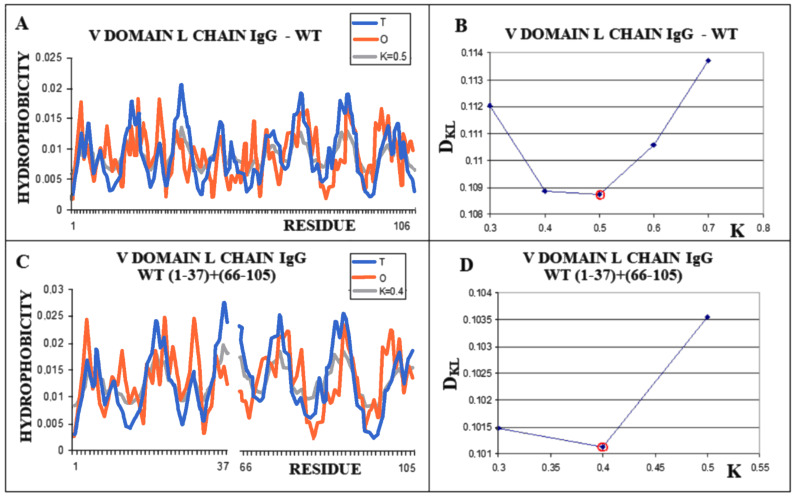
Structural characteristics of the V domain of the IgG light chain in its WT form. (**A**)—Profiles of T-navy blue, O-red, and M-gray for the complete chain. The M distribution is obtained for the parameter K = 0.5. (**B**)—Change in the *D_KL_* value for the O–M relation with different values of the K parameter. The lowest value was indicated as representing the shortest distance. (**C**)—Profiles of T-navy blue, O-red, and M-gray for the WT form of V domain hover limited to fragments present in amyloid form. The M distribution is obtained for the parameter K = 0.4. (**D**)—Change in the *D_KL_* value for the O–M relation with different values of the K parameter. The lowest value was indicated as representing the shortest distance between the O and M distribution.

Similarly, the V domain of the IgG light chain (Figure 4) shows the best fit of the O distribution to the M distribution for K = 0.5 for the complete domain. For sections of the chain present in amyloid form (1–37, 66–105), this value is K = 0.4.

**Figure 5:**
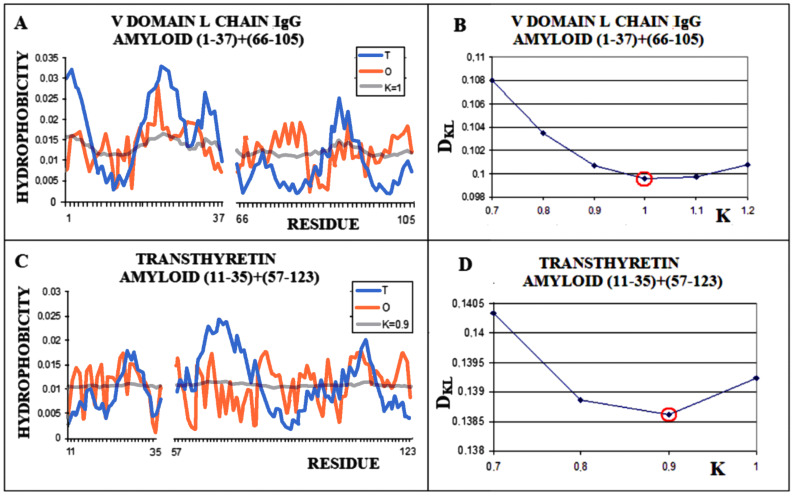
Characteristics of amyloid forms: (**A**)—Distributions of T-navy blue, O-red, and M-gray for a single-chain as appeared in amyloid form. (**B**)—Change in the *D_KL_* value for the O–M relation with different values of the K parameter. The lowest value was indicated as representing the shortest distance between the O and M distribution. (**C**)—Profiles of T-navy blue, O-red, and M-gray for a single-chain structure of transthyretin in the form present in amyloid. The M distribution is obtained for the parameter K = 0.9. (**D**)—Change in the *D_KL_* value for the O–M relation with different values of the K parameter. The lowest value was indicated as representing the shortest distance between the O and M distribution.

The analogously determined status of a single chain—the amyloid fibril component composed of the chain of the V domain of the IgG light chain—is obtained for K = 1.0 (Figure 5A,B). For the transthyretin amyloid fibril, an optimal match of the O and M distribution is obtained with K = 0.9 (Figure 5C,D).

In both examples discussed, the K value increases significantly from the native form to the amyloid form. This means that the structure of the environment (the structure of the external environmental field), while changing, favors a different folding of the chain from the native, which guarantees its biological function.

**Figure 6:**
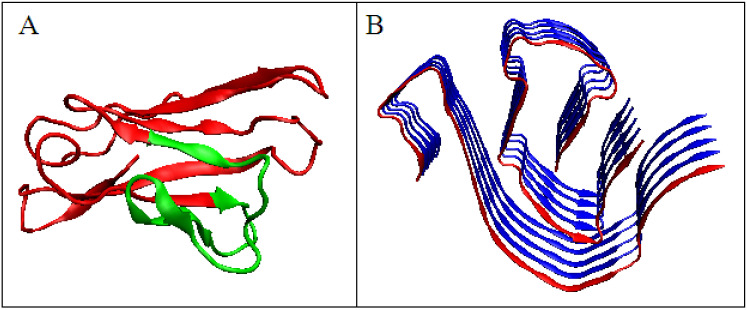
Domain V light chain of IgG. (**A**)—Native form, the red fragment present in amyloid form. (**B**)—Amyloid form, the red chain presents the form of the red fragment in A.

The analysis of the M distribution in Figure 5C reveals a significant similarity of the M distribution to the R distribution (R distribution is always parallel to the horizontal axis). The uniform distribution of the level of hydrophobicity within the protein can be interpreted as a kind of “vacuum folding”. Protein does not receive any signals from the outside, constituting its own environment. Nevertheless, the representativeness of the distribution M is apparent for the distribution O, which, when averaged, actually gives a nearly uniform distribution. The presentation in 3D form shown in Figure 6 visualizes the structural organization of the discussed fragments in both conformations: WT (Figure 6A) and amyloid (Figure 6B).

### 2.3. Amyloid Transformation, Accompanied by a Decrease in the Value of the Parameter K

**Figure 7:**
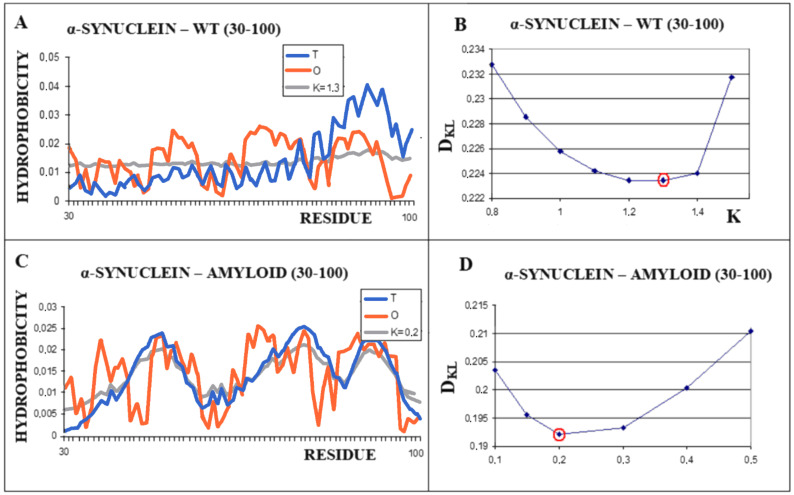
Characteristics of α-synuclein (**A**)—Distributions of T-navy blue, O-red, and M-gray for the structure of the 30–100 α-synuclein as is appears in WT form. (**B**)—Change in the *D_KL_* value for the O–M relation with different values of the K parameter. The smallest value K = 1.3 was indicated as representing the shortest distance between the O and M distribution. (**C**)—Profiles of T-navy blue, O-red, and M-gray for the structure of the fragment 30–100 of a single chain of α-synuclein in the form present in amyloid. The M distribution is obtained for the parameter K = 0.2. (**D**)—Change in the *D_KL_* value for the O–M relation with different values of the K parameter. The smallest value was indicated as representing the shortest distance between the O and M distribution.

A different scenario seems to take place for the protein α-synuclein. The amyloid form (fragment 30–100) of this protein takes the value RD = 0.473 for the T-O-R relationship. This means the presence of a clearly marked hydrophobic core and a decreasing level of hydrophobicity as you move away from the center. It is not possible to determine the RD parameter for the complete system (chains 1–140 do not interact with other chains due to the very loose structure of sections 1–30 and 100–140). These loose chain fragments form an additional intermediate zone between the marked hydrophobic core (as indicated by RD <0.5) and the polar surroundings. The transformation of the native biologically active form into the amyloid form is associated with a significant decrease in the value of the parameter K from the value of 1.3 for the native structure to K = 0.2 for the form present in the fibril (Figure 7). The juxtaposition of the T and O profiles and the optimal M distribution for the part of the α-synuclein chain present in amyloid form (lane 30–100) reveals a fit of the distributions for the determined K values.

**Figure 8:**
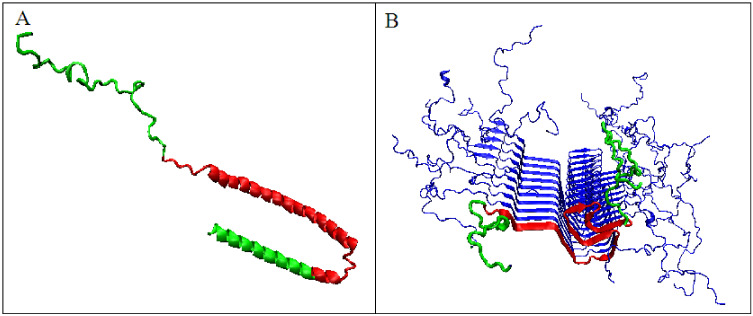
3D presentation of α-synuclein. (**A**)—Micelle-bound form, red fragment distinguished to show the fragment building the amyloid form of this protein; green fragments do not participate in fibril formation. (**B**)—Amyloid form, red fragment – amyloid form, green fragments – not participating in the construction of fibril.

The significant decrease in the K parameter value for α-synuclein suggests the following scenario. In native conditions, α-synuclein has an imposed structure through a target-type molecule (presynaptic terminals) as a frame in which the protein’s matched structure exhibits a high RD value, since this protein does not represent a globular form with a marked core (Figure 8A). In the case of the experiment leading to obtaining the structure available in PDB, this frame is the micelle on which the analyzed protein is based (α-synuclein micelle-bound [44]). This molecule, devoid of a stabilizing factor for its biologically active form (ensuring that it performs this function), undergoes the process of free folding, which in the case of α-synuclein leads to a form consistent with that assumed in the fuzzy oil drop model (Figure 8B). This interpretation is suggested by the change in the value of the parameter K = 1.3 for the complexed form with the target to the very low value K = 0.2 in the water environment. This means that in the case of a biologically active form, the influence of the environment forcing a given structure is expressed with a value of as much as K = 1.3, which exceeds the assumed maximum level of influence equal to 1. The presence of such a significant modification of the aquatic environment reveals a significant factor influencing the formation of α-synuclein structure under the conditions guaranteeing a biological function.

The native structures of the amyloid Aβ (1–42) protein are not available. It is known, however, that it is naturally a component of the integral transmembrane amyloid precursor protein (APP). The biologically active structure (performing its role) probably shows a high K value. The chain structure adapted to the target probably—similar to α-synuclein—does not show a globular structure. Therefore, the hydrophobic core is probably not present. Observing the low K value for amyloid forms, one can speculate a scenario for the amyloid transformation of Aβ (1–42) similar to that proposed for α-synuclein.

The structures of the biologically active forms of Aβ amyloid are unknown. There are only reports of the presence of helical species in a membrane-mimicking environment or in aqueous TFE solutions [99,100,101].

## 3. Discussion

The detailed analysis of the status of amyloid forms is not possible without reference to the examples of other proteins, both intrinsically disordered proteins (prions) and non-complexing proteins. This comparison allows a conclusion regarding the specific status of the amyloid forms. The summary of the K parameter values for the discussed proteins allows for the distinguishing of two scenarios for the amyloid transformation. One is the significant increase in the K parameter (natural water polar environmental impact) for the amyloid form. The status of this group of proteins in the WT form is defined by much lower values of the RD parameter. This means that proteins in their native form acting in an aqueous environment represent a structure similar to that formed by directing the polar water field. These proteins fulfill specific biological functions; hence, the RD values exceed the level of 0.5, showing a local mismatch between the O distribution and the T distribution [36]. Therefore, the native form does not present the status expressed as RD <0.5. A detailed analysis of the immunological domains and their specificity presented in [102] indicates a specific adaptation of the V domains to the function performed. Similarly, transthyretin also shows a local, well-defined, specific mismatch in the distribution of T and O [103,104,105]. The amyloid transformation associated with a significant increase in the value of the K parameter (Table 1) indicates the need for an external factor modifying the nature of the environment that favors the structural transformation leading to the formation of the amyloid form. In this transformation, there is also another modification of the FOD model—FOD-A (A stands from amyloid)—which consists of changing the external force field favoring the formation of a globular structure in the form of 3DG into the 2D Gaussian (2DG) form, as all the amyloid fibril structures known so far show a flat two-dimensional structure with an active center marked, but with reference to the 2DG form [106]. This means favoring the decay of the sigmaZ parameter by approaching zero. In this interpretation, a significant place is occupied by the presence of the secondary structure, which is the β-structure. Helical forms are not able to generate flat, two-dimensional forms. The helix is a typical three-dimensional arrangement. Shaking as a technique for obtaining the amyloid form may favor such a system, as the interphase structural form necessarily prefers a two-dimensional system of ordering. Here, an analysis of water structuring is needed in the event of an increase in the proportion of the water–air interphase form. Shaking is nothing more than increasing the presence of water–air interphase, and such an interphase certainly favors a 2D structuring of water. This significant change of the structuralization can be supported by the postulated need for a fundamental structural change in amyloid transformation [107]. Additionally, the favored β-structural organization fits very well with the 3DG to 2DG structural transformation [106].

This group of amyloid proteins also includes (speculatively) the tau protein. The premise is the high K value for the amyloid fibril of this protein.

The second group, unfortunately, is represented only by the α-synuclein protein. It is a protein with a high K value in the WT form, while the amyloid form has a surprisingly low RD value and a low K parameter value. This means that the protein in the WT form does not adopt a structure resulting from self-folding under the water environment, which should favor the formation of a globular form with a clearly marked hydrophobic core. The structure of the α-synuclein protein deposited in PDB is described by the authors as “micelle-bound human α-synuclein”, which means that this protein requires the presence of a specific target structure to perform its biological activity [44]. A structure fitted to a “target” is an “incapacitated” structure. Adaptation to the “target” rules out the folding mechanism that is favored by contact with the aquatic environment. Similar conclusions can be drawn from the analysis of membrane proteins, where the differences in the status of the domains anchored in the membrane as compared to domains remaining in the cytoplasmic environment show similar differences to those observed in the discussed example of α-synucleins. Protein devoid of a specific frame that maintains a forced structural form (high K value and high RD value) results in a significant difference to the distribution typical of the aquatic environment with a centric hydrophobic core and a polar surface. A protein lacking a frame that maintains this forced form behaves like any other protein in the aquatic environment. It should be noted that the structure of the amyloid generated by the complete α-synuclein chains shows a status of RD <0.5, and the part with the amyloid fibril alignment (Table 1) shows a value very little above the level of 0.5. This means that amyloid, in this particular case, arises as a result of a natural process leading to the formation of a structure acceptable to the aquatic environment.

The amyloid forms of the Aβ (1–42) proteins whose native structure is not known in the amyloid form show very low K values, similar to α-synucleins. Literature reports claim that they are derived by sequential proteolytic cleavage of the integral transmembrane amyloid precursor protein (APP). Therefore, there is a high probability that their native structure is the result of a significant share of the target molecule acting as a frame. Depriving this frame creates a structure resulting from the standard influence of water molecules on the structure of proteins [108]. The status of the protein with a high RD value for the T-O-R relationship should also be considered. A high value means that the O distribution is close to the R distribution. The R distribution represents a status devoid of diversification in the hydrophobicity distribution depending on the location in the protein. Such a status is interpreted as devoid of the influence of the external field, both polar (water) and hydrophobic (membrane). Unified distribution in the entire protein body can be interpreted as a situation analogous to a specific “vacuum”. In relation to the protein, it can be interpreted that the protein itself constitutes the field that determines its folding. An exception to the proposed model and interpretation is the example of the tau protein [43], whose partial characterization (native form is not available) suggests the scenario proposed for the transthyretin and V domain of the IgG light chain. An analysis of the structure in a biologically active form would be a very valuable source of information.

Information-theory-based considerations provided two scenarios for the amyloid transformation mechanism [109].

The introduction of the concept of information in relation to the assessment of the structure of proteins is due to the fact of their specificity. It requires a unique structural form to encode the selective recognition of the interaction partner—the substrate, in the particular case of enzymes. In this system, the structure of an idealized micelle with a surface covered with polar groups shows a preference for the interaction with water (apart from ionic interactions). This applies to both the spherical and ribbon-like micelle structures. Therefore, the micelle with spherical structure presents a low level of information encoded. Similarly, a ribbon-like micelle represents a comparable status. The ideal micelle is treated as a null record because it shows no record other than what would arise from the preference for interacting with water.

**Figure 9:**
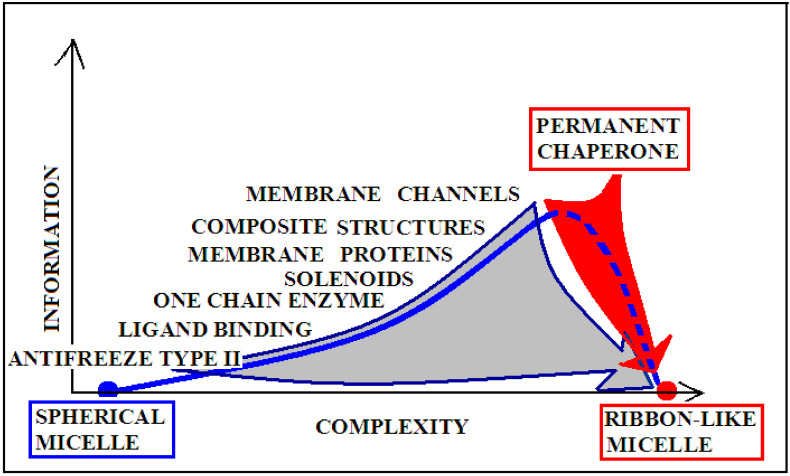
Graphical presentation of the relationship of the degree of complexity of the protein structure and the amount of information encoded in the structure that guarantees a specific biological activity. Proteins with a spherical micellar structure determined by sequence and a ribbon-like micellar form show the lowest demand for external information. The aquatic environment is a sufficient source of information. The more complex the structure (complexes), the greater the information expenditure. The position of “permanent chaperone” defines the status of a protein that fulfills its biological role only by interacting with the appropriate organelles. The absence of the “partner” results in a change of structure solely due to the influence of the aquatic environment (red arrow). The gray arrow shows the transformation forced by the change of the environmental factor (shaking) resulting from the experiment. Dashed line—pathological process.

The degree of complexity of recording the specificity and also the form of the information sent back to the environment (most likely affecting the local structuring of water) is significantly varied, as shown in Figure 9. For example, an enzyme may represent the order of the micelle-like hydrophobicity distribution, with the exception of ordering or rather lack of ordering within the active center. This form of local disorder is a form of recording information to enable specific interaction with the substrate. It seems that the amount of information contained in structures that require constant interaction with another system (cytoskeleton systems, membranes, etc.) is the highest. This is due to the dependence of a given structure on the constant presence of a “partner”. Its loss leads to structural changes directed exclusively by the aquatic environment. Such a polypeptide chain freed from the solid permanent chaperone adopts a structure resulting from the interaction with water. Therefore, in the case of the structure of the natural form of α-synuclein, the value of K is high, and after adjusting the structure to water conditions, the value of this parameter decreases. The red arrow on Figure 9 visualizes the process occurring after losing contact with the “permanent chaperone”, which ensures the high-information structure revealing the biological activity. Reaching the structure directed by the water environment deprives the protein of information coded in a biologically active form. 

The gray arrow in Figure 9, however, presents the possibility of converting any protein into the amyloid form in vitro, which is equivalent to the loss of specific information coded in a biologically active forms of these proteins. Reaching the form of amyloid is equivalent to represent the information-deprived structural form.

The term “complexity” expresses the complication of building a given structure. The lower “complexity” is attributed to the structure of the one-chain classical micelle, which arises spontaneously, shows high symmetry, and possesses no encoded form of “information” (vertical axis). The micelle, due to the lack of any form of differentiation, is unable to recognize the molecule that interacts with it. A single-chain enzyme that does not fully restore the micelle structure (shows local incompatibility with the micelle-like system) presents a value of RD greater than 0.5. The elimination of two catalytic residues and one Cys constituting the disulfide bond results in obtainment of a value of RD below 0.5, which means achieving the micelle-like status. This means that the protein encodes the “information” that makes it possible to recognize a specific substrate. An example of such a protein is lysozyme (see Supplementary Materials S5—Miscellaneous proteins). Therefore, a single-chain enzyme is ranked higher in the “complexity” (RD > 0.5) and higher in the “information” scale—expressed in the possibility of recognizing a specific substrate. The need for proteins to generate quaternary structure places these proteins on the “complexity” scale at the higher levels. At the same time, such structures must be the carriers of “higher” information, because apart from the specific activity (which they mostly represent), they must have information about the method of generating the structure of the complex. From the point of view of complexity, membrane proteins are quite different in relation to the soluble proteins. Their high “complexity” on the hypothetical scale results from the need to build a structure, for example of the beta-barrel type, fulfilling additionally the condition of exposure of hydrophobic residues on the surface (contrary to the soluble proteins). The recording of this phenomenon is associated with the need to record more information. On this scale, the highest positions are intended for proteins requiring the presence of a “permanent chaperone”. The structure of these proteins depends on the external “information” in the form of an appropriate frame, which determines the correct biological function. This dependence on an external partner makes these proteins highly demanding in terms of both complexity and information. This relationship was described in detail in [109]. Work on the quantitative assessment of this relationship is ongoing and will be available in the near future.

**Figure 10:**
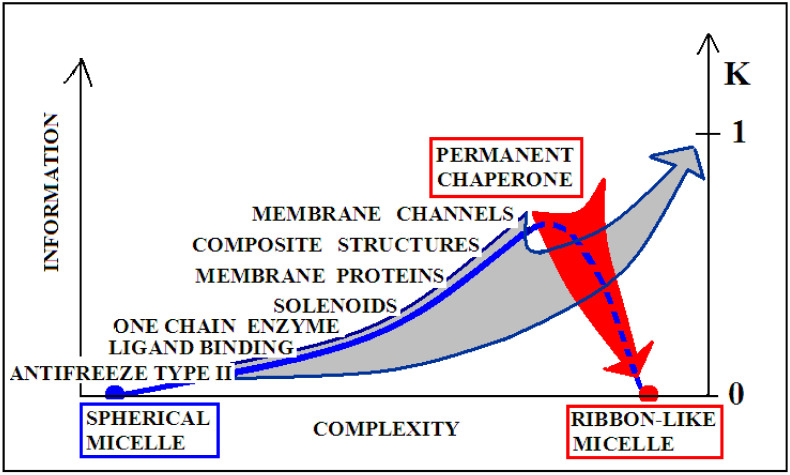
Graphical presentation of the structure complexity relation to the K coefficient expressing the participation of the modified environment—the presence of factors favoring the amyloid transformation.

Figure 10 visualizes two scenarios based on the discussion of the K-scale parameter value. The red arrow visualizes the pathological process taking place in vivo, where a protein devoid of a “permanent chaperone” takes a structural form resulting from the influence of the polar aquatic environment. Despite the distribution consistent with 2DG distribution, it generates a concentration of higher hydrophobicity in the central part of the proto-fibril or super-fibril [106].

On the other hand, the gray arrow illustrates the process in which a micelle-like protein folded in the water environment undergoes a transformation under the influence of environmental changes leading to a state with a high K value. The source of this is, for example, shaking used in in vitro techniques (or other chemical factors like pH, ionic strength, presence of other molecules, etc.). This environment leads to the adoption of a forced structure with a high proportion of K. Factors supporting the amyloid transformation in vitro—according to the FOD model—introduce the changes in water structuralization rather than immediately influence the structure of the polypeptide chain. The probability of global structural changes of the polypeptide chain as the effect of immediate local interaction with chemical compounds is very low. The influence on the protein structure by single chemical compounds is observed very often; however, the basis—keeping the 3DG form—remains in those cases. The changes of structuralization of the water environment treated as an external force field is able to globally influence the “philosophy” of folding, which fits to the active participation of water treated as a continuous force field.

Of course, the presence of amyloid transformation may also be alleged in vivo for both IgG light chain [110] and transthyretin [111,112]. However, they are often associated with the presence of mutations [113,114].

The exception in the given set is the tau protein, which breaks out of the scheme presented as a proposed interpretation of the amyloid transformation. The tau protein is highly hydrophilic compared to the rest of the proteins discussed here. The mean intrinsic hydrophobicity for the proteins discussed in the present work is 0.542, while the tau sequence shows a value of 0.455 on the scale used for the calculations presented here. This difference makes the tau protein also rate as highly polar in experiments [115]. Therefore, this protein requires an in-depth analysis to clarify the specifics of this protein.

Dimerization is not necessarily a preliminary step for amyloid transformation. The formation of the dimer most likely preserves the 3DG organization according to a mechanism known to other complexes that form a new shared hydrophobic core. The multimers keep the 3D Gauss structuralization, while amyloid transformation requires significant structural changes, which according to our proposal are expressed by the change to 2DG organization. Shaking WT α-synuclein and its mutant form produces fibrils only for the native form [116]. The importance of the presence of solid–liquid and air–liquid interfaces for insulin aggregation kinetics has been identified as critical [117].

Dimers produced by the mutant form usually retain the 3DG system, creating a connection generating a new hydrophobic core still in the 3DG system. On the other hand, the form of WT undergoes this radical change [107] by transforming into a system based on 2DG organization. The formation of a 3DG dimer blocks the way to polymerization, creating an entropy-friendly system for contact with a water. In the case of transthyretin, the elimination of 23 (out of 123) residues (resulting in RD value > 0.5) reveals a part of the dimer with a Gaussian 3D distribution stabilizing the dimer structure, as shown in [118]. In the case of the amyloid structure, elimination does not solve the problem, as it would concern the essence of ordering. Obtaining the RD < 0.5 status for the amyloid form requires the exclusion of a chain segment along the entire length of the fibril, which is—in contrast to globular proteins—a disruption of the entire fibril structure.

A perfect example of the influence of the environment on shaping the structure of a protein is a set of two proteins, the structure of which depends on the immediate vicinity of the lipid layer and the detergent [119]. The beta-barrel ordering depends on the size of the amphipathic neighbor. The size of the detergent micelle limits the range of ordering compatible with it. In the case of the bi-layer lipid system, the contact range with the hydrophobic system is wider, and the beta-barrel arrangement involves a much larger part of the chain.

Recently identified and discussed polymorphic forms of amyloid are very interesting material for the analysis presented here [120,121]. The K parameter is expected to vary depending on the polymorphic structure.

## 4. Materials and Methods

### 4.1. Data

Table 3 presents the proteins analyzed in this work. The list presents other amyloid proteins, prions, proteins recognized as intrinsically disordered, and short peptides related to amyloids. The miscellaneous proteins are also discussed to put the analysis of amyloid-related proteins in the wider context of proteins not complexing. It allows interpretation of results concerning amyloids discussed formerly [122].

In order to broaden the number of examples of proteins representing a wider spectrum of applications of the FOD model and its modified form, FOD-M, the analysis also contains the following proteins representing other structural groups, including amyloids, which are described in the Supplementary Materials. The first group designated by S1 in Supplementary Materials: 2KJ3 [50], 2LBU [51], 2MUS [52], 6EKA [53], 6LNI [54], 6UUR [55], 6VPS [56], 6ZCH [57], 6ZCF [57], 6ZCG [57], 5W3N [58]; group of prion proteins designated as S2 in Supplementary Materials: 1B10 [59], 3HAK [60], 3HER [60], 3HES [60], 1QLX [61], 2XK3 [62], 2XKU [63], 1I4M [64], 5YJ5 [65], 6FNV [66], 6HEQ [67]; the group of intrinsically disordered proteins designated as S3 in Supplementary Materials: 2L42 [68], 1RX9 [69], 2LPB [70], 1LMW [71], 1CK9 [72], 1AGQ [73], 1U96 [74]; short peptides designated as S4 in Supplementary Materials: 1OEH [75], 2IV4 [76], 1S4T [77], 2IV6 [78], 1OEP [79], 1M25 [80], 2RMW [81], 2RMV [81], 1YJO [82], 6PQA [55], 5K2G [83], 6CLx * [84], 3NHC [85], 6PQ5 [55], 4ELH [86], 4ELI [86], 4W5x * [87]; and non-complexing proteins (arbitrarily selected) designated as S5 in Supplementary Materials: antifreeze: 1AME [88], 1EWW [89], 1MSI [90]; down-hill proteins: 2L6G [91], 2L6R [92], 1W4E [93]; fast-folding: 1W4K [93], 1WXC [94]; titin: 1TIT [95]; enzyme, lysozyme: 1LZ1 [96]; repressor: 1CMB [67]; transmembrane protein: rhodopsin 1AP9 [98] (* denotes the few structures differing by last letter in the PDB code system).

### 4.2. Description of the FOD-M Model

A short description of the fuzzy oil drop (FOD) model introduced earlier and used many times, as well as its modification—the FOD-M model—that takes into account the influence of the non-polar environments in protein folding, will be presented here. A detailed description of the FOD model can be found, for example, in two books [122,123].

The observed hydrophobicity (called *O*) $H_{i}^{O}$ in a particular position of effective atom of *i-th* residue (i.e., the average position of atoms that make up a given amino acid), being the result of the interactions with the surrounding residues, is calculated according to the Levitt equation [124], Equation (1):

(1) $$H_{i}^{O}=\frac{1}{H_{sum}^{O}}\sum_{j}\left\{\begin{array}{l} \left(H_{i}^{r}+H_{j}^{r}\right)\left(1-\frac{1}{2}\left(7{\left(\frac{r_{ij}}{c}\right)}^{2}-9{\left(\frac{r_{ij}}{c}\right)}^{4}+5{\left(\frac{r_{ij}}{c}\right)}^{6}-{\left(\frac{r_{ij}}{c}\right)}^{8}\right)\right)\;for\;r_{ij}\leq c\\ 0,\;\;for\;r_{ij}>c \end{array}\right.$$

The hydrophobicity $H_{i}^{O}$ collects the hydrophobic interactions in distance-dependent form, as given in the above formula with the cutoff distance (*c*) assumed according to the original work, 9Å. The denominator $H_{sum}^{O}$ (the sum of all $H_{i}^{O}$) makes the value in a normalized form. The $H_{i}^{r}$ and $H_{j}^{r}$ express the intrinsic hydrophobicity of the *i*-th and *j*-th residues, which can be taken according to the arbitrarily selected scale.

On the other hand, the theoretical hydrophobicity $H_{i}^{T}$ is expressed by the value of the 3D Gaussian function in a position of a given effective atom Equation (2):

(2) $$H_{i}^{T}=\frac{1}{H_{sum}^{T}}\exp\left(\frac{-{\left(x_{i}-\bar{x}\right)}^{2}}{2\sigma_{x}^{2}}\right)\exp\left(\frac{-{\left(y_{i}-\bar{y}\right)}^{2}}{2\sigma_{y}^{2}}\right)\exp\left(\frac{-{\left(z_{i}-\bar{z}\right)}^{2}}{2\sigma_{z}^{2}}\right)$$

The values for the $\sigma_{x},\sigma_{y},\sigma_{z}$ parameters are determined for the structural form individually according to the size and shape of protein under consideration.

The distributions of the observed hydrophobicity (*O*) and the theoretical hydrophobicity (*T*) as defined above, can be quantitatively compared using the divergence entropy $D_{KL}$ introduced by Kullback–Leibler [125]. To interpret the *D_KL_,* another reference distribution is used. The reference distribution is the uniform one R, where each residue is assigned the same hydrophobicity $R_{i}=1/N$, N being the number of amino acids in a polypeptide chain. Such a distribution was chosen to represent a distribution lacking any variation in the hydrophobicity within a molecule. A comparison of the $D_{KL}$ values for the relation O|T and O|R shows which “distance” is closer. The $D_{KL}$ values for T|O less than those for O|R allow one to infer the presence of a centric concentration of hydrophobicity and, thus, the presence of a hydrophobic core. To eliminate the necessity of using two values, the following parameter *RD* (Relative Distance) was introduced Equation (3):

(3) $$RD=\frac{D_{KL}(O|T)}{D_{KL}(O|T)+D_{KL}(O|R)}$$

The *RD* parameter expresses the degree of adjustment of the hydrophobicity distribution observed in a given structure, resulting from the distribution of residues with a specific intrinsic hydrophobicity to the idealized distribution expressed by a 3D Gaussian function spread over the folding chain at a given moment of the folding process.

The values of *RD* < 0.5 indicate the presence of the hydrophobic core generated during the folding process.

The modification of the FOD model, the so-called FOD-M model, extending the participation of a non-polar environment in protein folding relies on introducing the structural specificity of membrane proteins. In the membrane proteins, an exposure of hydrophobic residues on the surface is expected. The channel function requires the presence of polar residues in the center (i.e., in the place of a channel’s course). Therefore, the hydrophobicity distribution (*M*) in such a membrane protein is “inverted” to the centric one and can be expressed by the function Equation (4):

(4) $$M_{i}=T_{MAX}-T_{i}$$

where $T_{MAX}$ is the maximum value in the determined theoretical distribution T created according to the 3D Gaussian function.

The T distribution is modified by assigning a status to individual residues in the form of complements to the values expected for the centric distribution. According to the assumptions, the distribution expressed above should meet the conditions present within the membrane protein. However, it turns out that the omnipresence of the aquatic environment also imprints the structure of the membrane protein. Therefore, the external field directing the protein-folding process turns out to be some form of the consensus between the centric field and the inverted one M and can be expressed as Equation (5):

(5) $$M_{i}={\left[T_{i}+{\left(T_{MAX}-T_{i}\right)}_{n}\right]}_{n}$$

where index *n* denotes normalization. The distribution expressed by the above equation defines the influence of the membrane environment (“inverted”) in the extreme case, which is the membrane being a fully hydrophobic environment.

The *K* coefficient was additionally introduced to make the above definition universal Equation (6):

(6) $$M_{i}={\left[T_{i}+K{\left(T_{MAX}-T_{i}\right)}_{n}\right]}_{n}$$

The *K* coefficient expresses the consensus between the water environment (centric hydrophobic core) and the hydrophobic environment of the membrane. This consensus does not have to take the extreme form, as in the case of a membrane protein with an ion channel present. The value of the *K* coefficient is assumed to be in the range 0 < *K* < 1. The values close to 0 represent the proteins with a high degree of centric hydrophobicity. The values of *K* close to 1 express structures with a significant portion of the membrane environment. It also turns out that the value of the *RD* parameter is highly correlated with the value *K*. Both these values express the degree of deviation from the micelle-like hydrophobicity distribution within the protein. However, while the *RD* parameter states the fact (and its strength) that the distribution differs from the centric distribution, the value of *K* measures the participation of other-than-polar factors influencing the folding process.

Therefore, for each structure, apart from the value of the *RD* parameter, the optimal value of *K* is determined by searching for such a distribution *M* that results in the minimal distance $D_{KL}$, Equation (7):

(7) $$D_{KL}\left(O|M\right)=\overset{N}{\underset{i=1}{\sum }}O_{i}\log_{2}\frac{O_{i}}{M}$$

from an *O* distribution.

It is expected that the values of parameter *K* may be protein specific. Values in the range 0 < *K* < 1 are interpreted as the degree of participation of the factor disturbing the basic environment, which is a polar aquatic environment. The value of *K* expresses the degree of the need to correct the T distribution. The higher the value of *K*, the higher the modification of the target distribution for the distribution of *O*. At the same time, the value of *K* expresses the degree of involvement of other-than-water factors in the formation of the structure of the protein folding in the environment characterized by the distribution of *M*. The recent analysis of many different proteins allows identification of proteins characterized by *K* > 1.0 (even *K* > 3.0). These are the objects of a current analysis, which will be published soon.

**Figure 11:**
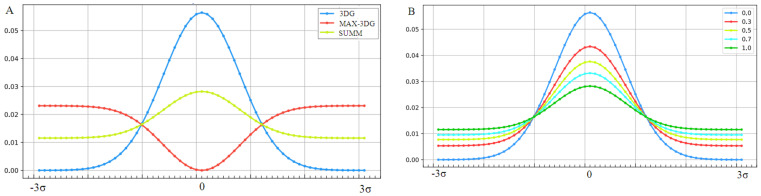
Changes in the form of the external force field that determine the environmental characteristics of the folding protein. (**A**)—Successive stages of force field modification: blue line, field expressed by 3D Gauss distribution (3DG); red line, “inverted” field preferring the exposure of hydrophobic residues on the surface (MAX-3DG); green line, resultant field for the derived field consensus from water and medium is the disturbing field (SUMM) for K = 1. An example of the application of this type of modification was proven on the example of membrane proteins operating in a hydrophobic environment. (**B**)—Gradual “weakening” of the structure of the hydrophobic core (decreasing concentration of hydrophobicity in the central part) with a increasing value of the parameter K, which expresses the strengthening influence of the disturbing factor.

The graphic presentation of the influence of the presence of the “inverted” field on the hydrophobicity distribution shown in Figure 11A shows the influence of considering the factor disturbing the distribution resulting from the exclusive participation of the polar water field. Figure 11B shows a decrease in the maximum level of hydrophobicity concentration with a simultaneous increase in levels in the area close to the protein surface, depending on the value of the *K* coefficient.

### 4.3. Programs Used

The program for calculation of the RD parameter is accessible on the GitHub platform: https://github.com/KatarzynaStapor/FODmodel (accessed on 27 August 2021).

The program VMD was used for 3D presentation of the discussed structures [126].

## 5. Conclusions

The modified FOD-M model was validated previously on the basis of membrane proteins [49]. The modification introduced in the FOD-M model with the parameter K, taking into account other environmental factors, allows the assessment of the status of amyloid proteins from the point of view of the participation of these external factors. This assessment was the main purpose of the work. Observations of changes in the values of the parameter K when switching to the amyloid form—according to the two opposite scenarios—suggest an analogous classification of amyloid formation based on the criterion of environmental influence, which in the FOD-M model is expressed by the parameter K.

As a result of the analysis, it is possible to identify the presence of two mechanisms leading to the formation of amyloid fibrils. This fact results from the different role played by these proteins. Some of them are soluble (like transthyretin or V domain light chain of IgG) and work in the aquatic environment. The second group consists of proteins that in their active form remain in complexes with organelles or cells, mainly of the nervous system. This solid form of the complex has been defined as a “permanent chaperone” that maintains the appropriate active structural form of the protein. The amyloid transformation of soluble proteins requires a significant change in the environment, which prefers a structure different from the physiological one. Shaking as an in vitro form accompanying amyloid transformation favors the 2DG arrangement with respect to structural forms preferring the formation of a hydrophobic core. However, the 2DG structure of the chain represents the exposure of the hydrophobic surface (central part of the disc). This exposure causes the fibril formation, which according to this mechanism may be continued infinitely.

In the case of proteins losing the permanent chaperone, guaranteeing biological activity, they adopt the structure directed by the water environment. Their sequence must be specifically prepared to generate the structure to fit the target (neuronal organelles). Looing the target, the protein adopts the structure as the individual molecule accepts the structure directed by the water environment. Since the sequence is not prepared to generate the globular structure, the chain adopts the structure as far as possible to follow the conditions generated by water (exposure of polar parts with hydrophobic parts hidden in the center). It turns out the chain is not able to adopt globular (3DG) structure, and it generates the 2DG structural form, which, however, requires complexation to minimize the exposure of hydrophobicity. This is why the ribbon-like micelle is generated [106]. The very large size of amyloid fibrils makes them insoluble and thus very demolishing for cells structure and function.

The presence of the environment and the specificity of its impact on protein folding in the generation of protein complexation and its biological activity have been demonstrated in [121,122].

These two types of transformation were identified by determining the K coefficient, which expresses the degree of participation of the environment (other than water) in shaping a given structure. This coefficient expresses the degree of participation of the non-aquatic environment affecting the stabilization of the appropriate spatial structure. The large values of K express the strong influence of the environment (shaking preferring the 2DG orientation). Small K values suggest adaptation to the water environment.

The simulation of the protein-folding process should be performed in an environment expressed by the distribution of 3DG and in an environment that gradually changes through an increase in the K value. As shown in [37,49], the K value turns out to be specific for a given protein. Further analysis of proteins for the corresponding K parameter value is performed on a large database.

The significant contribution of the air–water and oil–water interphase in the drug complexation process underlines the participation of the environment in this process [30]. A similar dependence of structural changes conditioned by environmental characteristics was shown in the case of insulin aggregation [8,115].

The very origin of neurodegenerative diseases seems to be the presence of the factor which causes the dissociation of proteins addressed to be complexed with specific organelles. These proteins, left as independent molecules, adopt the structure directed by the water environment. The formation of amyloid deposits based on the V domain of light chain of IgG and transthyretin requires significant interference from the environment other than water. This speculation can be drawn on the basis of the analysis presented in this paper.

The importance of the aquatic environment is increasingly recognized in folding/unfolding processes [127,128].

In the context of the fuzzy oil drop model, the widely discussed role of intrinsically disordered proteins (IDP) in amyloid transformation does not appear to play a critical role, due to the local nature of chain fragments, while amyloid transformation is global [129,130,131,132,133,134]. For the analysis of the amyloid transformation based on the fuzzy oil drop model, the characterization of the down-hill and fast-folding protein structures is important because these examples confirm the validity of the assumptions of this model [135,136,137,138,139] (see also Supplementary Materials, S5, Miscellaneous proteins).

## Figures and Tables

**Table 1:** List of proteins for which the K values corresponding to the smallest distance (*D_KL_*) of the O distribution to the modified M distribution were determined. Parameter values were determined by regarding single chains as structural units (a 3D Gaussian function was generated for a single chain in a fibril).

PROTEIN	WT				AMYLOID			
	PDB-ID	RD T-O-R	RD M-O-T	K	K	RD M-O-T	RD T-O-R	PDB-ID
Transthyretin—A	1DVQ	0.562	0.342	0.5				
(11–35) + (57–123)	1DVQ	0.584	0.331	0.5	1.8	0.242	0.757	6SDZ
IgG – VL	4BJL	0.547	0.390	0.5				
(1–37) + (66–105)	4BJL	0.516	0.405	0.4	1.1	0.229	0.755	6HUD
TAU					1.2	0.323	0.674	5O3L
α-Synuclein	1XQ8	0.643	0.340	1.3				
(30–100)	1XQ8	0.672	0.319	1.3	0.5	0.377	0.568	2N0A
Aβ(1–42) (15–40) D23N					0.5	0.349	0.626	2MPZ
Aβ(1–42) (1–40) E22Δ					0.7	0.334	0.629	2MVX
Aβ(1–42) (11–42)					0.5	0.385	0.538	2MXU

**Table 2:** List of proteins for which K values corresponding to the smallest distance (*D_KL_*) of the O distribution to the modified M distribution were determined. Parameter values were determined by regarding proto- and/or super-fibrils as units (a 3D Gaussian function was generated for proto-fibril and super-fibril).

PROTEIN	WT				AMYLOID			
	PDB-ID	RD T-O-R	RD M-O-T	K	K	RD M-O-T	RD T-O-R	PDB-ID
Tranthyretin - A	1DVQ	0.562	0.342	0.5				
(11–35) + (57–123)	1DVQ	0.584	0.331	0.5	1.1	0.305	0.694	6SDZ(F)
IgG – VL	4BJL	0.547	0.390	0.5				
(1–37) + (66–105)	4BJL	0.516	0.405	0.4	1.0	0.193	0.793	6HUD(C)
TAU					1.2 1.2	0.334 0.259	0.664 0.728	5O3L superfibril
α-Synuclein	1XQ8	0.643	0.340	1.3				
(30–100)	1XQ8	0.672	0.319	1.3	0.2	0.430	0.506	2N0A(E)
Aβ(1–42)(15–40)D23N					0.4 0.2	0.496 0.457	0.554 0.491	2MPZ(M) superfibril
Aβ(1–42) (1–40) E22Δ					0.8 0.5	0.326 0.315	0.649 0.567	2MVX(E) superfibril
Aβ(1–42) (11–42)					0.3	0.436	0.515	2MXU(F)

**Table 3:** A set of the described proteins along with a brief description of the analyzed fragments. A brief description of the proteins in question is also provided.

PROTEIN	PDB-ID		Fragment in Amyloid	Refs.
	WT	Amyloid		
Transthyretin	1DVQ – A	6SDZ	(11–35) + (57–123)	[38,39]
IgG – VL	4BJL –VL	6HUD	(1–37) + (66–105)	[40,41]
Tau		5O3L		[42]
α-synuclein	1XQ8	2N0A	(30–100)	[43,44]
Aβ (1–42)		2MXU	(11–42)	[45]
Aβ (1–42)		2MPZ	(15–40) D23N	[46]
Aβ (1–42)		2MVX	(1–40) E22Δ	[47]

## Data Availability

All data can be available on request addressed to corresponding author. The program allowing calculation of RD is accessible on GitHub platform: https://github.com/KatarzynaStapor/FODmodel (accessed on 27 August 2021).

## Supplementary Materials

The following are available online at https://www.mdpi.com/article/10.3390/ijms221910587/s1.
