# Supplementary material for: In Silico Modeling of the Influence of Environment on Amyloid Folding Using FOD-M Model
Source: Int J Mol Sci. 2021 Sep 30;22(19):10587. doi: 10.3390/ijms221910587 (PMC8508659; doi:10.3390/ijms221910587)
Supplement: Supplementary file 1 [file ijms-22-10587-s001.zip › ijms-1377391-supplementary.pdf]

SUPPLEMENTARY MATERIALS

The results presented here are intended to enable the comparison of the proteins discussed in the main part of the work with others related to the amyloid transformation process as well as not related. The spectrum of proteins characterized by the parameters K from K = 0 up to K ≈ 1 is presented.

As this is a completely new description of the protein structure, access to the characteristics of other proteins seems to be needed.

The following protein groups are included in this section:

S1 – other amyloids not discussed in the main part

S2 – prion proteins

S3 – intrinsically disordered proteins

S4 – peptides identified as fragments of amyloid chains with a large part directing the structural transformation of amyloid protein chains

S5 – miscelaneous proteins – for parameter K in the range: 0 < K < 1.0

The presence of the analysis of these proteins was suggested by the Reviewers in order to allow a broader interpretation of the RD and K parameters used in the main part of the paper.

The S1 group contains examples to highlight the variability in the characteristics of proteins with known amyloid structure. However, their biologically active version is not known.

The S2 group contains examples of proteins that play an important role in amyloid transformation.

Group S3 contains examples of proteins with an identified form of IDP (intrinsically disordered proteins) including the entire protein molecule as well as chain fragments showing such characteristics.

The S4 group are peptides identified in searching for the causes of amyloid transformation. Their characteristics given here allow to assess the predisposition of a given protein fragment to generate a soluble structure (globular with a central hydrophobic core, especially in the case of complexes of many peptides).

Group S5 are representatives of proteins not associated with the phenomenon of amyloid transformation. Representatives of proteins with K = 0 , i.e. the down-hill, fast-folding proteins are shown. Also the titin domain is presented, which represents the order status highly consistent with the micelle-like form (although for this protein the value of K = 0.2). Characteristics of the enzyme representative - lysozyme, which is characterized by the presence of a local incompatibility of the T and O distributions is also given. The location of the region of incompatibility is closely related to the enzymatic activity of this protein. The list of miscellaneous proteins is closed with the example of a membrane protein, where the use of the 1-3DG model is justified due to the specificity resulting from the location of this protein in the membrane.

All groups were characterized in the same way giving a set of values of RD and K parameters in order to facilitate the interpretation of these parameters. Exemplary sets of the T and O distributions and the optimal M distribution for the determined optimal value of the K parameter are also given. In addition, the selected examples of structures are shown in the 3D form, with the emphasis on protein regions important for the discussion of the considered model, which visualize the features important for the discussed phenomenon.

S1 Other Amyloids

The list of amyloids currently discussed supplements the previous analysis. Its purpose is to treat the analysis presented above in a broader context.

Table S1.1 List of amyloid forms and their characteristics. The status of the whole fibril and the single chain treated as an independent structural unit are given. "R" in the right-hand column indicates the status of the modified distribution which is close to the R distribution.

PDB ID	T-O-R	K	M-O-T

2KJ3	fibril	0.535	0.5	0.375	3 chains
	chain A	0.618	0.6	0.318	79 aa
2LBU	fibril	0.698	0.8	0.247	5 chains
	chain A	0.652	0.8	0.323	71 aa
2MUS	super-fibril	0.535	0.5	0.389	5 chains
	Proto-fibril	0.503	0.4	0.410	71 aa
	chain A	0.636	0.8	0.337	71 aa
6EKA	fibril	0.708	0.9	0.266	5 chains
	chain A	0.661	0.7	0.309	53 aa
6LNI	super-fibril	0.794	2.3	0.205	**R 2 proto-fibrils**
	proto-fibril	0.810	2.2	0.189	**R 5 chains**
	chain A	0.663	0.8	0.285	60 aa
6UUR	fibril	0.745	1.1	0.230	5 chains
	chain A	0.730	0.8	0.248	40 aa
6VPS	fibril	0.790	1.3	0.203	3 chains
	chain A	0.806	1.1	0.193	31 aa
6ZCH	fibryl	0.562	0.6	0.357	6 chains
	chain A	0.585	0.5	0.336	69 aa
6ZCF	fibril	0.620	0.8	0.358	6 chains
	chain A	0.756	1.6	0.243	37 aa
6ZCG	Super-fibril	0.778	2.0	0.220	**R**
	Proto - Fibril	0.623	0.9	0.356	6 chains
	chain A	0.673	0.9	0.308	37 aa
5W3N	fibril	0.698	0.8	0.247	9 chains
	chain A	0.748	0.7	0.207	46 aa

The results summarized in Table S1.1 allow for speculation on the degree of the influence of environmental factors. It turns out that the vast majority of amyloid forms require high values of K to obtain the optimal distribution for a given structure. High values of K suggest the need for a significant modification of the T distribution to map the O distribution. Only 2MUS amyloid shows relatively low values of both RD and K, which would suggest that the fibrillar system arose spontaneously with the support of the aqueous environment (the proto-fibril structure shows a very exeeding of cut-off RD = 0.5 - similar to the Alpha-synuclein structure assessment). The examples where the value of K exceeds the level of 2.0 deserve attention. Such a high modification of the T distribution leads to a distribution M similar to the R distribution. The R distribution is characterized by a uniform distribution without any differentiation in the levels

of hydrophobicity within the molecule (complex). It means a state corresponding to some kind of "vacuum" and thus isolating oneself from any external influences. Neither the polar aquatic environment (3D Gauss) nor the hydrophobic environment (TMAX - 3DG) affects the decomposition of hydrophobicity in the system. The system decides about itself without taking into account the influence of external factors. This is the case with 6LNI for the super- and proto-fibril forms. Single chains as fibril constituents obtained this status in the case of 6ZCF.

Exemplary distributions for the chains representing the discussed group of proteins are shown in Figure S1.1, and 3D structure on Figure S1.2.

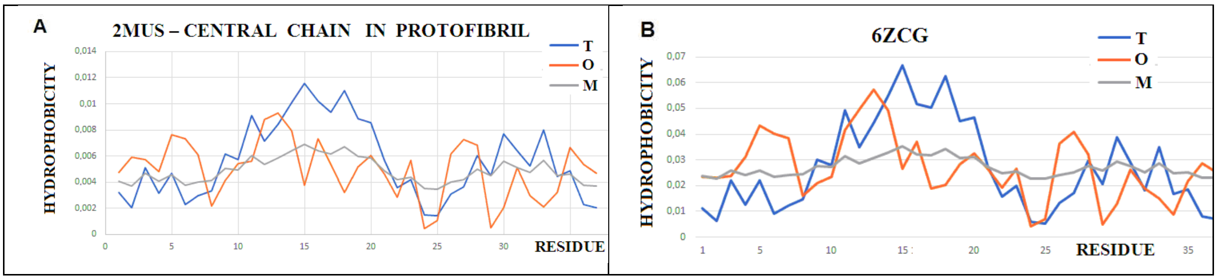

Figure S1.1 The distributions of T (blue), O (red) and M (gray) determines the status of the chain - a component of: A - the 2MUS amyloid protofibril. The M distribution was obtained for K = 0.4 as the optimal value for protofibril; B - 6ZCG protofibril. Optimal K value **= 2**

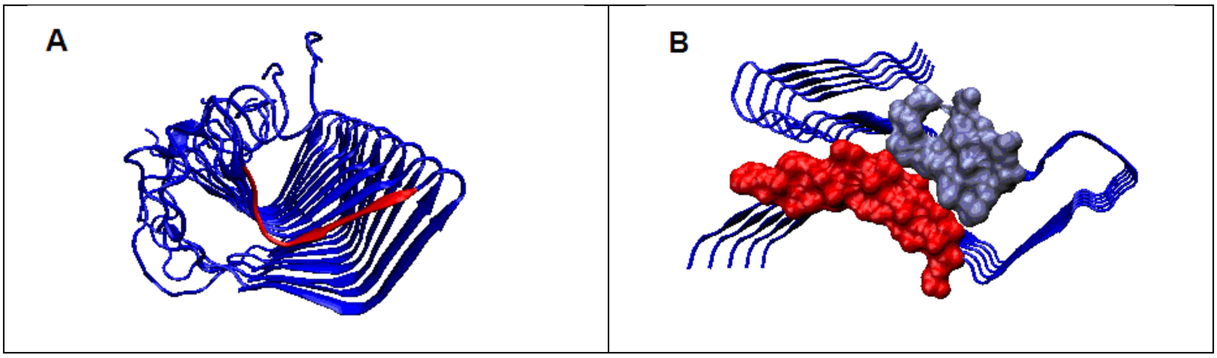

Figure S1.2. 3D presentation of :A – 2MUS – red fragment participate in hydrophobic core generation (positions 10-20). B – 6LNI – red fragment (35-42) discordant versus expectation to participate in core generation, iceblue fragment (6-15) participates in core generation

S2 Prions

The inclusion of prion proteins complements the considerations of the mad mechanism of amyloid transformation. The characterization of these proteins based on the fuzzy oil drop model has already been presented in [102].

Table S2.1. Characteristics of prions under consideration

PDB ID	T-O-R	K	M-O-T	
1B10	0.429	0.3	0.415	104 aa

3HAK		0.461	0.3	0.398	103 aa - membrane
1QLX		0.387	0.2	0.440	104 aa
2XKS		0.383	0.2	0.441	99 aa
2XKU		0.455	0.3	0.422	94 aa
1I4M		0.670	1.0	0.319	108 aa – membrane
5YJ5 –	chain	0.539	0.5	0.376	152 aa – membrane
	Fragment	0.476	0.3	0.397	???
6FNV	chain	0.583	0.5	0.354	140 aa
	fragment Beta	0.471	0.3	0.410	
6HEQ		0.543	0.5	0.375	122 aa
3HEQ	A+B	0.738	1.5	0.258	Homo-dimer
	chain A	0.519	0.4	0.362	102 aa
	chain B	0.496	0.4	0.373	102 aa
3HER	A+B	0.717	1.5	0.280	homo-dimer
	chain A	0.458	0.3	0.405	97 AA
	chain B	0.486	0.4	0.380	97 AA
3HES	(A+B)	0.702	1.3	0.290	homo-dimer
	chain A	0.505	0.4	0.368	98 aa
	chain B	0.462	0.3	0.402	98 aa

The prion characterization results show the presence of a micelle-like hydrophobicity order with low RD values for the T-O-R relation and low values of the K parameter (Figure S2.1).

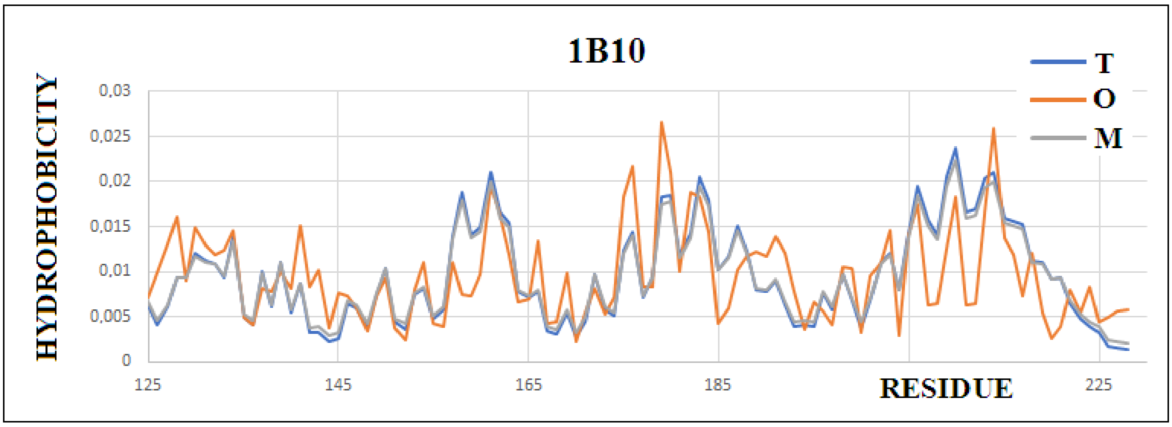

Figure S2.1. The distribution of T (blue), O (red) and M (gray) for the prion representative - 1B10. Visible significant adjustment of the distribution of T and O and a slight modification expressed with the value of K = 0.3

This suggests the generation of the structures of the proteins in question as spontaneously compatible with the influence of the aqueous environment preferring the centric concentration of hydrophobic residues with a polar shell.

The dimer status, on the other hand, seems to be controlled by external - environmental factors. The P-P interface status in the three dimers listed in Table S2.1 is expressed by the RD values for the T-O-R relationship: 0.635, 0.681 and 0.539 for 3HEQ, 3HER and 3HES, respectively (Figure S2.2). These values indicate that the chain interface did not generate a common hydrophobic core. Thus, based on the fuzzy oil drop model the dimerization mechanism does not rely on complexation with the use of hydrophobic interactions. The domain-swapping dimerization mechanism in some cases consists in generating a common core by two chains with an interface status expressed as RD < 0.5 [103]. This is not the case here.

Low values of RD for single chains listed in Table S2.1 suggest the need for a significant contribution of environmental changing factors in the process of amyloid transformation. From the point of view of the ordering of the monomer structure, there are no factors destabilizing the tertiary structure, taking into account the presence of a hydrophobic core and additionally the presence of a disulfide bond [104].

The visualization of the distributions identified in the exemplary representatives of the prion group (Figure S2.1) for 1B10 reveals a high adjustment of the distribution of T and O and a slight modification represented by the distribution of M for the value of K = 0.3.

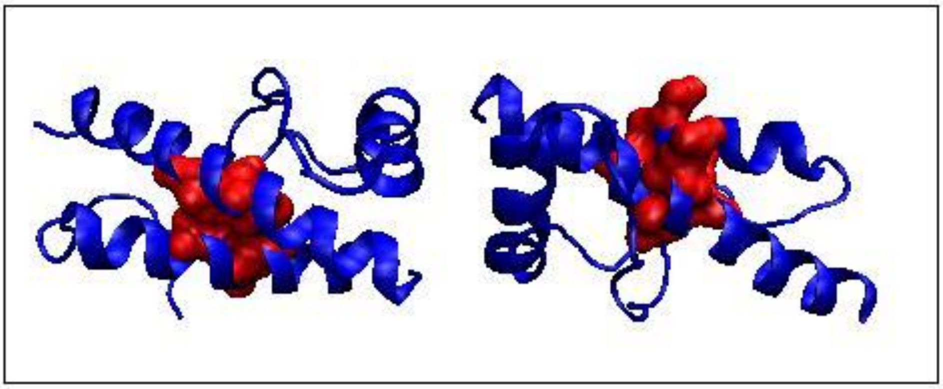

Figure S2.2. 3D prezentation of 3HER dimer. Residues distinguished in red participate in hydrophobic core construction in monomer. No common hydrophobic core in dimer.

S3 Intrinsically disordered proteins

These proteins have already been discussed in [107], where it was shown that often the absence of secondary structure in sections treated as intrinsically disordered does not mean a mismatch from the point of view of the structure of a common hydrophobic centric core. The same can be seen in several of the examples discussed here, such as in 2L42 or 1RX9, for example. A high degree of matching of the distribution of idealized segments identified as disordered is also visible - e.g. 1RX9 or 1CK9. In the case of 1RX9, the status of the interchain interface is expressed as RD = 0.337, which means that the residues building the interface contribute to the construction of a common hydrophobic core responsible for the stabilization of the complex.

Table S3.1. Characterization of a set of proteins with sections regarded as intrinsically disordered. Position "No" means the given fragment to be absent

PDB ID		T-O-R	K	M-O-T	Characteristics
2L42		0.387	0.2	0.481	DNA binding
1RX9		0.497	0.4	0.404	enzyme
	9-24	0.445			
	63-73	0.280			
	116-132	0.490			
2LPB	A+B	0.529	0.4	0.419	hetero-dimer
	Chain A	0.457	0.2	0.450	
	Chain B	0.713	1.4	0.284	
	B in complex	0.670			
1LWM		0.546	0.6	0.409	DNA binding
1CK9		0.546	0.6	0.409	ribosomal protein l30
	16-29	0.379			
1AGQ		0.547	0.4	0.358	DNA binding
1U96		0.612	1.4	0.387	Chaperone
	No 1-27 fragment	0.516	0.4	0.468	

The interpretation of the results given in Table S3.1 reveals the differentiation in the status of the proteins with the intrinsically disordered fragments. Proteins with intrinsically disordered segments in their structures were selected as examples from the DISPROT database [108, 109].

The status of the protein with PDB ID 2L42 - DNA-binding protein RAP1 (Saccharomyces cerevisiae) turns out to be unique. The N-terminal domain available in PDB, which is fully recognized as disordetred, according to the fuzzy oil drop model shows a hydrophobicity distribution consistent with the assumed, i.e. expressed by the 3D Gauss function. In this structure - despite the low presence of secondary structure ordering - the value of RD < 0.3 suggests the presence of a hydrophobic core to a degree very close to the idealized distribution (the value of Rd < 0.3 is very rarely identified in the proteins studied so far). There is no disulfide bond in this domain. Of the two stabilization factors of the tertiary structure, it is the presence of a hydrophobic core that determines the stability of this domain. A very low value of K = 0.2 means that a slight modification of the order of T provides an optimal distribution - a scheme that reproduces the actual structure of the domain in question. This is also confirmed by the low value of RD for the M-O-T relation.

A similar situation is observed in the case of 1RX8 - dihydrofolate dehydrogenase (EColi), where a centrally located hydrophobic core with a polar coating is present. The segments 9-24 and 63-72, identified in the DisProt database as disordered show a perfect adjustment of their hydrophobicity distribution to the idealized expected distribution. Hetero-dimer 2LPB – a

central activation domain of gcn4 bound to the mediator co-activator domain 1 of gal11 / med15. Two chains of different chain length (chain A - 81 aa, chain B - 34 aa) represent different statuses. Chain A shows an order according to a micelle-like distribution. The entire B chain is identified as disordered in its entirety. A lower RD value expressing the status of this chain in the complex suggests the adoption of a structural form that matches the form of this chain to the structure of the complex.

1LWM - DNA binding protein shows cavity of large size in relation to the size of the whole molecule. Therefore, the value of RD for the T-O-R relationship takes a high value > 0.5. Modification of the optimal distribution requires the value of K = 0.6, which means the need for a fairly significant influence of the factor disturbing the formation consistent with the centric hydrophobic core.

Protein with the identifier 1CK9 - yeast ribosomal protein l30 (Saccharomyces cerevisiae) shows a status of RD > 0.5, although the status of the segment mentioned by DisProt is surprisingly consistent with the expected distribution. The reason for the high value of RD results from the need to adapt the structure to a very complex structure in the conditions of biological activity of this protein as a ribosm component.

Relatively high values of the discussed parameters are present in the 1AGQ protein - Glial cell-derived neurotrophic factor (Rattus norvegicus). This is due to the presence of numerous disulfide bonds - three disulfide bonds are present in the 99 aa chain and one Cys is involved in the binding of SS to the Cys of the second chain. As shown in [110], the relationship between two factors stabilizing the tertiary structure, which are disulfide bonds and the presence of a hydrophobic core, tend to be compatible (the presence of disulfide bonds does not disturb the structure of the centric hydrophobic core) or contradictory (the system of disulfide bonds imposes a different status on the inter-Cys sections from the expected hydrophobic nucleus). The values of RD 0.452, 0.603 and 0.603 for the T-O-R relationship of the sections defined by disulfide bonds: (42-103), (69-132) and (73-134) respectively, suggest the arrangement of the chain fragment consistent with the idealized one only for the first disulfide bond.

In the discussed set of exemplary proteins, the chaperon 1U96 protein with the helix-hair-pin structure, both for the complete chain and for the ordered domain, shows a status deviating from the system with a centric hydrophobic core.

The exemplary 1RX9 protein reveals a clearly consistent status (T and O distribution) of the segments recognized as intrinsically disordered fragments. This illustrates a classification different from the secondary structure when adopting the criterion of participation in the structure of the hydrophobic core (Figure S3.1). The sections identified as intrinsically disordered are located mostly on the surface, forming part of the outer shell, showing a high match to the idealized distribution (Figure S3.1 and Figure S3.2. A).

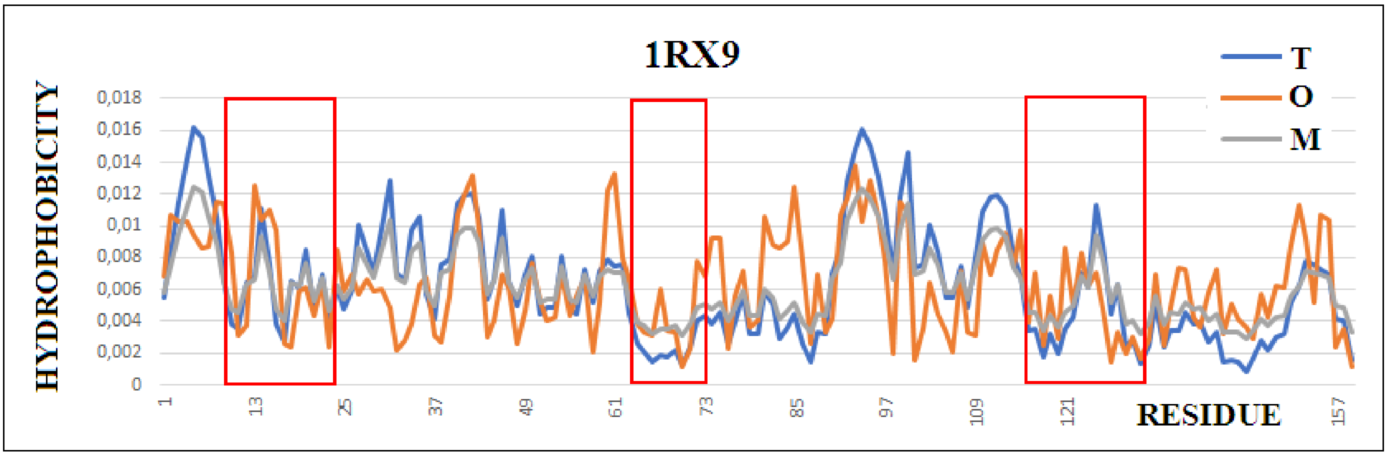

Figure S3.1. The distribution of T (blue), O (red) and M (gray) for a representative of proteins identified as intrinsically disordered (1RX9). The segments identified as intrinsically disordered are distinguished by boxes, which show a high adjustment of the distributions in these segments, expressed quantitatively in Table S3.1.

In the 2LPB complex, the status 0.414 of P-P interface means that the complex is formed by seeking to generate a nucleus centric system for the dimer. The state with RD < 0.5 is not reached, although the interface status indicates a favorable arrangement of interacting residues (Figure S3.2.B).

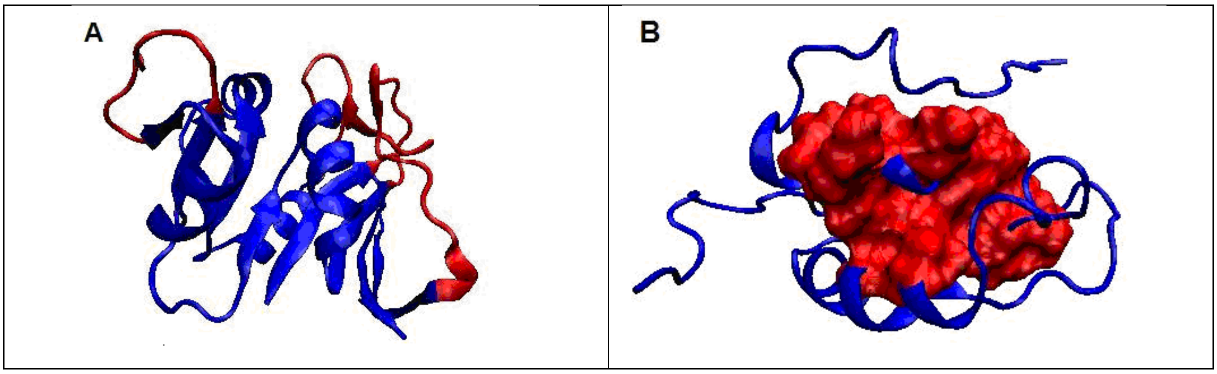

Figure S3.2. 3D presentation of : A – 1RX9, red residues – recognised as intrinsically disordered, however well accordant with hydrophobic core construction; B – 2L42 - red residues participate in hydrophobic core formation

Proteins with the intrinsically diosoredred status were the object of analysis using the fuzzy oil drop model. This analysis showed that these segments fit the distribution of a centrally ordered hydrophobic core despite the low ordering according to the second-order classification [111].

S4 Short peptides – amyloid related

The structure of some short peptides identified as components of proteins with tendencies for amyloid transformation is available in PDB. This makes it possible to track effects of order within the hydrophobicity distribution despite the small size of these objects. In this category, single short peptides as well as their complexes in the form of homo- or hetero-dimers as well as complexes with a greater number of chains are

discussed. They exemplify the differential status of these short peptides and their complexes. In this group, significant differentiation is observed with the range of RD values from 0.112 to 0.908. A low RD value for a low amino acid monomer indicates the presence of a hydrophobic residue in the center of the chain for a linear structural form, or even such a short chain exhibits a local distortion from linearity resulting in a centrally located concentration of higher levels of hydrophobicity. This interpretation is not conclusive since the kernel can be identified in a chain with polar residues with lower polarity in the central part of the chain. However, this proves that highly polar residues remain exposed to the environment in competition with lower polarity residues.

On the other hand, dimers or complexes with a higher number of components clearly indicate that even such short chains that do not generate the tertiary form complex, minimizing the unfavorable entropy contact of hydrophobic residues in the polar environment of water. The hydrophobicity distribution in the short peptides was previously analyzed with the fuzzy oil drop model in the context of Congo red complexation [112].

Table S4.1. Characteristics of short peptides under consideration

PDB ID	T-O-R	SEQUENCE	Chain length
1OEH	0.470	HGGGWGQP	8 aa
2IV4	0.568	VNITIKQHTVTTTTKG	16 aa
1S4T	0.646		21 aa
2IV6	0.432		23 aa
1OEI	0.799		24 aa
1M25	0.639		26 aa
2RMW	0.415		26 aa
2RMV	0.376		26 aa
1YJO	0.396	NNQQNY(acy)	7 aa
6PQA	0.112	GAVVGG	6 aa
**5K2G**	**0.908**	GNNQQNY	7 aa
**6CLx**	**0.908 - 0.794**	GSNQNNF	7 aa
3NHC	0.357	$[GYMLGS]_2$	2x6 aa
6PQ5	0.109	$[AGAAAA]_2$	2x6 aa
	0.092 – 0.374	$[GSNQNNF]_2$	2x6aa
4E1H complex<br>Individual chains	0.246<br>0.213 – 0.279	$[HDCVNI]_6$ / $[EQMCIT]_6$	2-mer (6-mer)
4W5x	0.232 – 0.419	$[GGYVLGS]_7$	7-mer
4E1I	0.205 – 0.327	$[HDCVNI]_5$ / $[EQMCIT]_6$	Hetero-complex

Due to the difficulties in visualizing the status of short sections, representatives with extreme degrees of compliance of the T and O distributions were selected: a high degree of non-compliance - 5K2G (Figure S.4.1.A and Figure S4.2.B) and a high degree of compliance: 6PQA (Figure S4.1B and Figure S4.2.A). Representatives of the complexes formed by the discussed peptides 1OEH and 4E1I are also shown (Figure S4.1.C and S4.1.D).

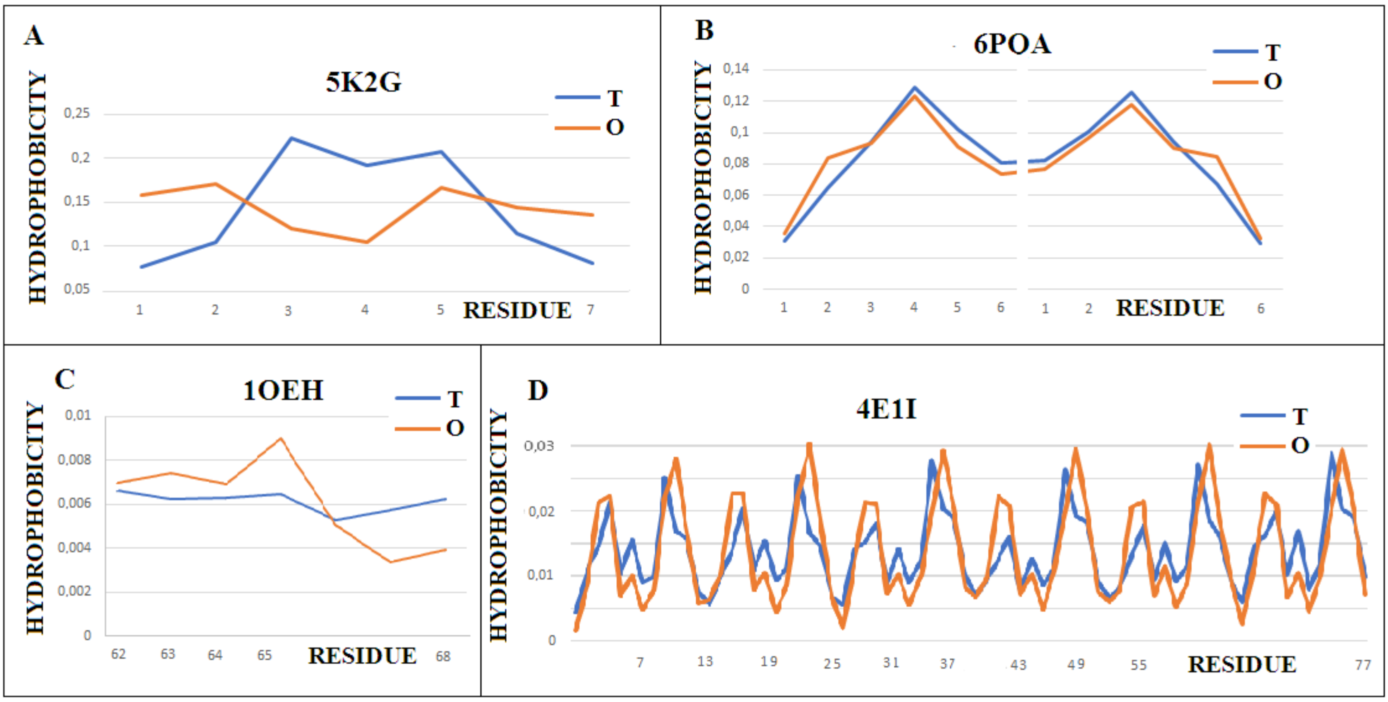

Figure S4.1. Distributions of T (navy blue) and O (red) for representatives of short peptides both in the form of single chains and complexes.

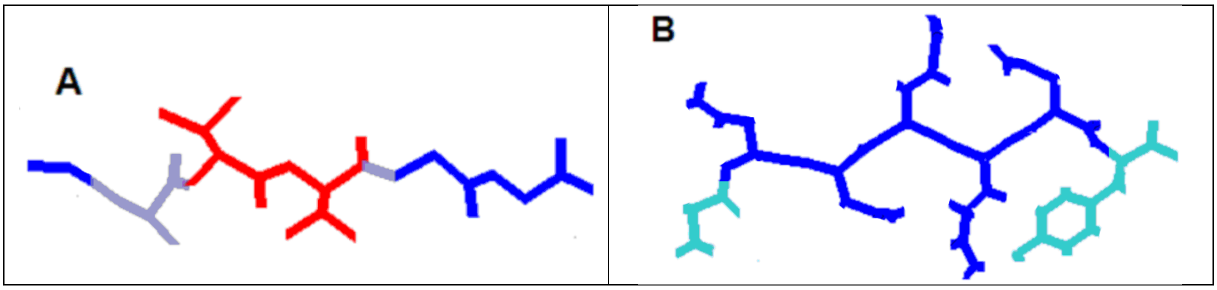

Figure S4.2. 3D presentation of short peptides: A – 6PQA – 6-residues peptide with hydrophobic residues in central part (red) and hydrophilic on N- and C-terminal positions (blue) – potentially ready to generate the complexes with central hydrophobic core present; B – 5K2G – highy polar residues with no possibility to generate the construction with hydrophobic core

S5 Miscellaneous proteins – non-complexing proteins

Soluble proteins with a globular structure are important for the analysis of structural changes. Protein complexation is considered as a result of the protein-

protein interaction can occur with the preservation of 3D Gauss ordering - observed in standard complexes - and complexing present in amyloids, where interchain contact is present on large stections of chains in the beta-structural ordering that prefers 2D Gaussian system. Classic non-aggregating proteins (in both forms of understanding this concept) are proteins from the down-hill, fast-folding or antifreeze type II and III groups. These proteins are discussed in detail in [35].

As the discussion of these proteins was carried out without taking into account the FOD-M model (modification based on the determination of the K parameter), a few selected examples are shown here. The summary of the results of the proteins described here (Table 4.1) shows a low degree of visibility of factors other than water (low K values) with RD for the T-O-R relation below 0.5. This summary indicates the aquatic environment as a source of information, which, together with the information carried by the amino acid sequences, constitutes a complete portion of information needed to adopt the structure represented by the FastFolding, downHill and antifreeze proteins, whose full solubility in the water environment determines their biological activity (coverage of proteins surface with a polar layer guarantee solubility and impose a structuring of water different from that of ice). This is expressed by the value of K = 0 for the aforementioned proteins. Antifreeze protein in the form of a solenoid shows a negligible need for the participation of modifying the standard environment, which is water.

The titin domain - taken as an example of a protein with a single step unfolding process [113-117] showing a status with a low value of the RD parameter also requires minimal environmental modification to obtain its structure. It can also be an expression of determinism inherent in a sequence for which a different, more optimal structure is not achieved. Similarly, a single-chain enzyme - lysozyme - showing the value of the RD parameter slightly above the adopted limit of 0.5 for proteins with a hydrophobic core, requires a slight modification of the external field (K = 0.5). Lysozyme is an example of a protein with a local defect in O distribution versus T. This mismatch is limited to the status of catalytic residues (35E and 53D) and exposed disulfide bond. Eliminating these residuals from the calculation of the RD parameter results in obtaining a value < 0.5 with a reduced modification of K = 0.4. An example of a protein representing the distribution expected by the amphipathic environment of the membrane is rhodopsin, the status of which is described by a high value of the RD parameter and a high value of the parameter K. This is obvious from the expectation of exposure of hydrophobic residues to the surface in order to have a favorable entropy state for contact with the membrane.

Table S5.1. Parameters of non-complexing proteins representing forms with high concordance of T and O distributions and membrane protein revealing the need to modify the FOD model to the FOD-M form.

PDB – ID	T-O-R	K	M-O-T	Characteristics
1AME	0.300	0.0		Antifreeze type III
1EWW	0.458	0.3	0.460	Antifreeze - solenoid
1MSI	0.320	0.0		Antifreeze
2L6G -	0.404	0.0		Down-Hill
2L6R - 1	0.399	0.0		Down Hill

1W4E	0.328	0.0		Fast Frolding
1W4K	0.369	0.0		Fast Folding
1WXC	0.356	0.0		Fast Folding
1TIT -	0.425	0.2	0.461	Titin
1LZ1 -	0.529	0.5	0.393	Lysozyme
	0.493	0.4	0.400	Lysozyme - No 35, 53, 128 aa
1CMB A+B	0.489	0.4	0.400	Homo-dimer
Chain A	0.568	0.6	0.384	
Chain A	0.506	0.4	0.425	Exposition on surface engaged in P-P interaction eliminated
1AP9	0.677	0.9	0.290	Membrane protein - rhodopsin

The proteins presented in Table S5.1 are intended to show the differentiation of the status of proteins, which turns out to be closely related to their function and - which is important from the point of view of the discussed issue - with the relation to the environment in which their biological activity is revealed.

To take into account also the description of the status of dimerizable proteins - Table S5.1 contains 1CMB homodimer in which the monomeric units show a disordered status from the point of view of the structure of the hydrophobic core. Their maladjustment consists in the exposure of hydrophobic residues on the surface, which interact in the complex, building a hydrophobic core common to the dimer, resulting in a structure representing the micelle-like form (Figure S5.1 and Figure S5.2).

In the list of proteins discussed here, rhodopsin is present as a representative of the membrane protein. Its presence aims to highlight the expressed characteristics by means of the discussed parameters, resulting from the specificity of the membrane protein. The differentiated (according to the fuzzy oil drop model assessment) status of the proteins is shown in Table S5.1 from the protein with a high degree of concordance of the T and O distributions (presence of an ordered hydrophobic core) - 1TIT, up to a membrane protein with exposure of hydrophobic residues on the protein surface - rhodopsin (1AP9), which requires significant modification of the target M (K = 0.9). The example of the 1CMB complex (present hydrophobic core) reveals a change in the monomer status by entering the dimer system (O distribution different from T distribution). The residues exposed to the surface in the monomer (unfavorable entropy effect) are part of the hydrophobic core in the dimer.

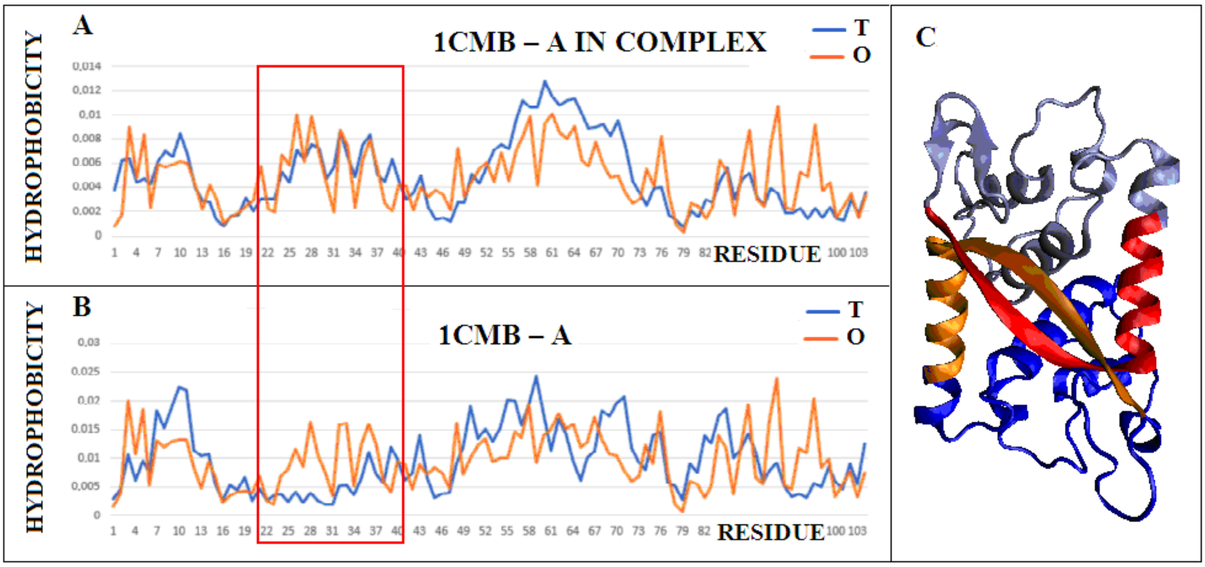

Figure S5.1. Distributions: T (blue) and O (red) A – chain A as a part of dimer; B – chain A as an individual structural unit revealing a change in the status of the section marked with a box, which in the structure of the monomer shows an excess of hydrophobicity, used to build the hydrophobic core; C - 3D presentation of 1CMB homodimer the red and orange fragments representing hydrophobicity excess in monomers create well constructed hydrophobic core in dimer (as shown in A and B)

In conclusion, it should be stated that the diversity of the values of RD and K parameters shown in the presented analysis indicates the specificity of the protein characteristics. Therefore, the interpretation of these parameters for proteins, the structure of which is available in both the WT and the amyloid versions, allows the use of RD and K parameters as a source of information on the type of structuring. Mainly the presence of environmental factors and their influence on the shaping of the appropriate structure seems to be expressed by the discussed parameters. The higher the values of RD and K parameters, the greater the importance of external factors.
